# Finding stable and closely linked QTLs against spot blotch in different planting dates during the adult stage in barley

**DOI:** 10.1038/s41598-024-51358-3

**Published:** 2024-01-08

**Authors:** Fakhtak Taliei, Hossein Sabouri, Borzo Kazerani, Shahram Ghasemi

**Affiliations:** 1 Department of Plant Production, College of Agriculture Science and Natural Resources, Gonbad Kavous University, Gonbad Kavous, Iran; 2 Department of Plant Breeding and Biotechnology, Faculty of Plant Production, Gorgan University of Agricultural Science and Natural Resources, Gorgan, Iran

**Keywords:** Biotechnology, Genetics, Molecular biology, Plant sciences

## Abstract

The common resistance to Spot Blotch (SB) and drought stress in barley was studied using a RILs population caused Kavir × Badia cross. These lines were inoculated with *Cochliobolus sativus* Gonbad isolate during the adult stage and were evaluated for three crop seasons in different planting dates. The different osmotic potentials during the flowering were regulated by changing the planting dates. In total, 43 lines had resistant to SB and drought. The high-density linkage map covered 1045 cM of barley genome. A total of five stable and closely linked QTLs to SB resistance were mapped on chromosomes 2H, 3H, 4H and 7H using genome-wide composite interval mapping. Moreover, four stable and closely linked QTLs to SB susceptibility were located on chromosomes 3H, 4H, 5H and 7H. Additionally, the ISJ19-A, SCoT7-C, ISJ17-B, Bmac0144k, iPBS2415-1, Bmac0282b and EBmatc0016 markers can be used for positive screening of resistant cultivars. However, ISJ3-C, UMB310, ISJ9-B, UMB706, D03-D and iPBS2257-A markers can be used for negative screening of susceptible cultivars in marker-assisted selection. The bioinformatics studies showed that *QRCsa-2H* (ISJ19-A region), *QRCsa-2H* (SCoT7-C-ISJ17-B region), *QRCsa-3H* (Bmac0144k region), *QRCsa-4H* (iPBS2415-1 region) and *QRCsa-7H* (Bmac0282b-EBmatc0016 region) are involved in the carboxypeptidase, Glycosyltransferase, transcription factors, kinase and AP2/ERF, respectively.

## Introduction

Spot Blotch (SB) or common brown spot disease is one of the common leaf disease of wheat and barley. SB is known as leaf blight and is vastly common in tropical regions worldwide^1^. The SB disease caused by *Cochliobolus sativus* (anamorph, *Bipolaris sorokiniana*) is the major foliar disease of barley; however also causes root rot, black point, seedling death and yield reduction^2,3^. Recently, this disease has been considered as one of the most important constraints of barley cultivation in warmer regions of the world. Nowadays, it has been tried to control the disease with IDM (integrated disease management) methods including the use of resistant cultivars, seed disinfection and crop rotation^4^.

In recent years, the ongoing phenomenon of climate change has given rise to a significant increase in both biotic and abiotic stresses, resulting in severe and irreversible damage to the environment and sustainable agriculture. Heat and drought stresses at the end of the growing season is one of the most important limitations of barley cultivation in hot and dry regions of Iran, especially the Gonbad Kavous region. In order to escape the cultivars from heat and drought stresses the end of the growing season, it is necessary to early planting date^5^. Therefore, drought stress during the reproductive stage can be simulated by adjusting the planting date and the effect of different levels of drought stress can be studied in plants. Notably, drought stress stands out as a particularly critical factor impeding the growth of small grain cereals worldwide^6,7^. This stress can significantly impact a plant's vital organs by promoting the generation of various types of reactive oxygen species (ROSs) at the molecular level. These ROS molecules play a pivotal role in the oxidation of crucial substances such as nucleic acids, proteins, and fats, ultimately leading to damage to the cell membrane and alterations in osmotic potential^8,9^. Drought stress has notable consequences at various stages of plant growth. During the seedling stage, it leads to a reduction in both germination rates and the lengths of plumules and radicles^10^. Moreover, when plants face drought stress in their adult stage, it results in a decrease in crucial agricultural traits. These include a decline in grain yield, 1000-grain weight, the number of filled grains, tiller count, panicle length, spikelet number, and fertility percentage. This stress also has morphological impacts, causing reduced plant growth and the development of dry, curled leaves^11^.

In the pursuit of sustainable agriculture and improved food security, it is crucial to identify sources of resistance to both biotic and abiotic stresses. This not only reduces the reliance on fungicides; however also contributes to environmental protection. In recent years, the process of identifying genetic resistance sources has often involved the use of bi-parental populations. These populations serve as a foundation for constructing a linkage map through the utilization of molecular markers. Subsequently, the technique of QTL (quantitative trait locus) mapping is employed to pinpoint regions associated with resistance and susceptibility to these stressors^12^. Additionally, these QTLs can be extrapolated to natural populations through the application of linkage disequilibrium (LD) and association analysis, further expanding our understanding of these crucial genetic traits^13^.

In different studies, the inheritance of resistance to SB is reported to be monogenic (oligogenic) or polygenic, and different resistance genes are expressed in the seedling or adult plant stage^1,14^. Under conditions without drought stress, some genes of resistance to SB including *Rcs1*, *Rcs2*, *Rcs3*, *Rcs5* and *Rcs6* were detected on chromosomes 2H, 1H, 5H, 7H and 1H, respectively^15^. Several studies have traced QTLs to SB resistance. In a study, SB disease from the source of three isolates including NO31, SH15 and SB61 was investigated on 449 barley accessions during the seedling and adult plant stages. Using 50 K single nucleotide polymorphisms (SNPs), genotyping was performed and a total of 38 MTAs (marker-trait associations) were traced by a genome-wide association study (GWAS) technique. Two major QTLs of resistance to SB were traced on chromosomes 1H and 7H. In addition, a novel minor QTL of susceptibility to SB was detected on chromosome 7H^16^. In another study, the seedling and adult plant resistance to SB disease was investigated on 3840 barley lines and cultivars. Using genome-wide association mapping (GWAM) technique, three QTLs of resistance to SB including *Rcs-qtl-1H-11_10764*, *Rcs-qtl-3H-11_10565* and *Rcs-qtl-7H-11_20162* were detected. Each of these QTLs alone reduced the disease level slightly; however, the combination of all three QTLs reduced seedling infection and adult disease severity (DS) by 47% and 83%, respectively^14^. In an experiment, the effect of SB disease was investigated on 261 barley genotypes in seedling and adult plant stages. The GWAM technique was performed using 13,182 PAVs (presence/absence variants) and 6311 SNP. In total, 23 QTLs in the seedling stage, and 15 QTLs in the adult plant stage were detected and some QTLs were common between the seedling and adult plant stages^1^. In another research, the effect of SB disease on 138 recombinant inbred lines (RILs) of barley was evaluated using 852 SNP. The QTL mapping technique traced a major *Scs6* gene of susceptibility to SB on chromosome 1H. Additionally, two QTLs of resistance to SB including *QSbs-1H-P1* and *QSbs-7H-P1* were detected on chromosomes 1H and 7H, respectively; these two QTLs were associated with the major *Rcs5* gene^17^.

Regrettably, within the Iranian barley germplasm population, there are no cultivars exhibiting a combination of resistance to SB disease and drought stress. To address this gap, it is essential to first create a population characterized by a high genetic potential for dual resistance to SB disease and drought stress. Subsequently, by identifying and isolating the transgressive segregated individuals and conducting supplementary studies, suitable cultivars can be developed. Moreover, once the QTLs responsible for resistance to these stresses are identified and confirmed, they can be effectively integrated into breeding programs, such as Marker-Assisted Selection (MAS).

The primary objective of this study is to assess and screen barley lines (using the Badia × Kavir cross) for their resistance to SB disease and drought stress during the adult growth stage. Additionally, we aim to pinpoint stable and closely linked QTLs as well as to elucidate the QTLs function in responding to SB disease and drought stress. Notably, the current study marks the first-ever attempt to trace common resistance QTLs in barley during the reproductive stage to SB disease and drought.

## Results

### Construction of a genetic linkage map

**Figure 1:**
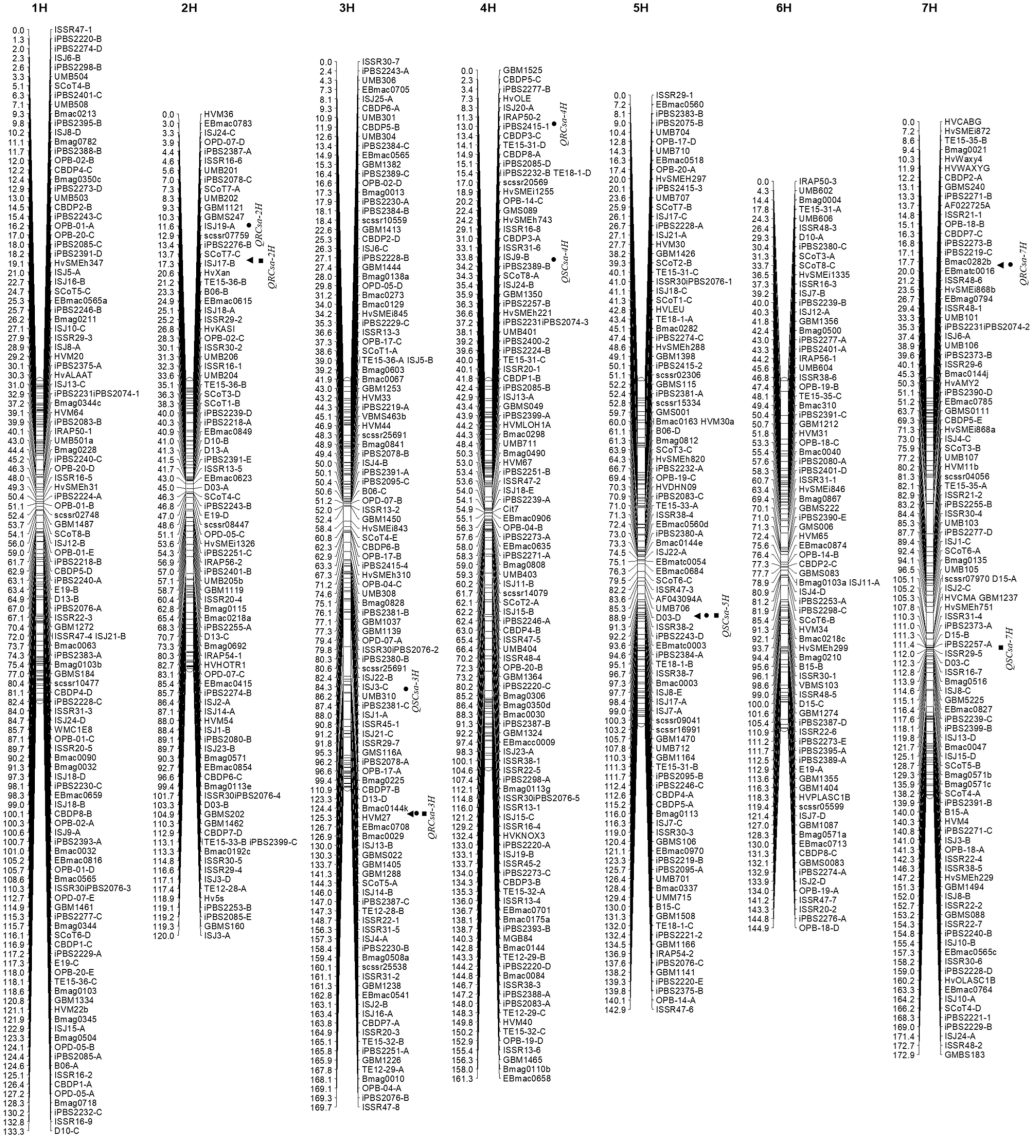
A high-density genetic linkage map based on co-dominant (SSR) and dominant (ISSR, CBDP, EST, SCoT, TE, RAPD, ISJ, IRAP, iPBS, iPBS-iPBS and ISSR-iPBS) markers using the RILs population from a cross between Kavir and Badia in barley (*Hordeum vulgare* L.). Additionally, the stable and closely linked QTLs of resistance/susceptibility against spot blotch and drought stress were mapped on chromosomes 2H, 3H, 4H, 5H and 7H. The triangle, circle and square symbols were indicated the early, conventional and delayed planting dates, respectively.

Molecular markers amplified 719 polymorphic alleles were located on seven chromosomes. In such a way that 120, 90, 114, 110, 100, 82 and 103 markers were detected on chromosomes 1H to 7H, respectively. The genetic map covered 1045.06 centi-Morgan (cM) of the barley genome and the average distance between two adjacent markers was 1.45 cM. The genome coverage was like this, chromosomes 1H to 7H covered 133.3, 120, 169.7, 161.3, 142.9, 144.9 and 172.9 cM of the genome, respectively (Fig. 1). The current genetic linkage map was almost saturated. Therefore, this high-density linkage map can locate QTLs on genome of barley.

### QTLs analysis in the early planting date or first planting date (PD1)

In the early planting date condition, the descriptive statistics (Supplementary Table 2) and Q–Q plot (Supplementary Fig. 1) showed that the AUDPC (area under the disease progress curve) trait in RILs population has normally distribute. Additionally, in this condition, the average phenotypic distribution of AUDPC in the studied population, including 103 F_8_ lines and parental cultivars was investigated (Supplementary Fig. 2). Moreover, in the early planting date, these results demonstrated the continuous and quantitative changes of AUDPC trait in SB disease. The high values ​​of the AUDPC trait indicated the susceptibility of the lines to SB disease and the low values ​​of this trait indicated the resistance of the lines to SB disease. In 2018/2019–2020/2021, some lines were more valuable than the Badia parent and some other lines were less valuable than the Kavir parent (Supplementary Table 2). The latter case indicated the occurrence of transgressive segregation phenomenon. This phenomenon is widely used in the breeding of quantitative traits^18^.

In the early planting date condition, 10 QTLs were identified on chromosomes 2H, 3H, 5H and 7H (Table 1). Two QTLs including *QRCsa-PD1-Y1-2H* (LOD = 3.38) and *QRCsa-PD1-Y2-2H* (LOD = 2.69) were detected on chromosome 2H with a marker distance of 15.49 cM in the stable region of SCoT7-C-ISJ17-B (Supplementary Fig. 7, Table 1). These QTLs were closer to the flanking marker SCoT7-C. The coefficient of determination of *QRCsa-PD1-Y1-2H* and *QRCsa-PD1-Y2-2H* were 14.20 (major) and 7.46 (minor), respectively. These QTLs have a negative additive effect, and thus, they inherited the SB disease resistance-enhancing allele from the Kavir parent. Also, in this condition, *QRCsa-PD1-Y1-2H* had the highest additive effect (-52.13) in the Kavir direction (Table 1). Two QTLs including *QRCsa-PD1-Y1-3H* (LOD = 3.76, R^2^ = 7.58) and *QRCsa-PD1-Y2-3H* (LOD = 2.83, R^2^ = 4.16) were with a distance 124.44 cM from the beginning of chromosome 3H in the stable region of Bmac0144k and were in the Kavir direction (Supplementary Fig. 7, Table 1). These minor QTLs were exactly mapped on Bmac0144k. In addition, *QRCsa-PD1-Y2-3H* had the lowest amount of genotypic variance (1006.83) in this condition (Table 1). Three QTLs including *QSCsa-PD1-Y1-5H* (LOD = 4.79), *QSCsa-PD1-Y2-5H* (LOD = 6.01) and *QSCsa-PD1-Y3-5H* (LOD = 5.33) were located on chromosome 5H with a marker distance of 87.10 cM in the stable region of UMB706-D03-D (Supplementary Fig. 7, Table 1). These QTLs were exactly between two flanking markers of UMB706 and D03-D. Furthermore, these QTLs had the highest LOD value in this condition. *QSCsa-PD1-Y1-5H*, *QSCsa-PD1-Y2-5H* and *QSCsa-PD1-Y3-5H* justified 21.70, 21.84 and 26.54 of the total phenotypic changes, respectively, and they were diagnosed as major. The positive additive effect of these QTLs showed that the allele that increased susceptibility to SB disease was inherited from the Badia parent. In this condition, *QSCsa-PD1-Y3-5H* had the highest additive effect (77.62) in the Badia direction (Table 1). Also, this QTL had the highest amount of genotypic variance (6024.99) in this condition (Table 1). Three QTLs including *QRCsa-PD1-Y1-7H* (LOD = 4.52), *QRCsa-PD1-Y2-7H* (LOD = 2.84) and *QRCsa-PD1-Y3-7H* (LOD = 4.00) were traced on chromosome 7H with a marker distance of 18.49 cM in the stable region of Bmac0282b-EBmatc0016 (Supplementary Fig. 7, Table 1). These QTLs were closer to the flanking marker Bmac0282b. The coefficients of determination for *QRCsa-PD1-Y1-7H*, *QRCsa-PD1-Y2-7H* and *QRCsa-PD1-Y3-7H* were 11.45 (major), 7.75 (minor) and 10.52 (major), respectively. Kavir’s parent alleles in these QTLs increased resistance to SB disease (Table 1).

**Table 1:** The stable and closely linked QTLs of resistance/susceptibility against spot blotch across multiple years in different planting dates of barley (*Hordeum vulgare* L).

Planting date	Year	QTL	Chr.	LOD	Position (cM)	AE^a^	Flanking marker	Marker type	Ϭ_g_^2^	R^2^	DPE^b^
PD1^c^	2018–2019	***QRCsa-PD1-Y1-2H***	2H	3.38	15.49	− 52.13	SCoT7-C, ISJ17-B	SCoT, ISJ	2717.64	14.20	Kavir
		*QRCsa-PD1-Y1-3H*	3H	3.76	124.44	− 38.10	Bmac0144k	SSR	1451.30	7.58	Kavir
		***QSCsa-PD1-Y1-5H***	5H	4.79	87.10	64.45	UMB706, D03-D	EST, RAPD	4154.26	21.70	Badia
		***QRCsa-PD1-Y1-7H***	7H	4.52	18.49	− 46.81	Bmac0282b, EBmatc0016	SSR	2191.49	11.45	Kavir
	2019-2020	*QRCsa-PD1-Y2-2H*	2H	2.69	15.49	− 42.51	SCoT7-C, ISJ17-B	SCoT, ISJ	1806.84	7.46	Kavir
		*QRCsa-PD1-Y2-3H*	3H	2.83	124.44	− 31.73	Bmac0144k	SSR	1006.83	4.16	Kavir
		***QSCsa-PD1-Y2-5H***	5H	6.01	87.10	72.74	UMB706, D03-D	EST, RAPD	5291.42	21.84	Badia
		*QRCsa-PD1-Y2-7H*	7H	2.84	18.49	− 43.32	Bmac0282b, EBmatc0016	SSR	1876.39	7.75	Kavir
	2020–2021	***QSCsa-PD1-Y3-5H***	5H	5.33	87.10	77.62	UMB706, D03-D	EST, RAPD	6024.99	26.54	Badia
		***QRCsa-PD1-Y3-7H***	7H	4.00	18.49	− 48.87	Bmac0282b, EBmatc0016	SSR	2388.10	10.52	Kavir
PD2^d^	2018–2019	***QSCsa-PD2-Y1-5H***	5H	4.98	87.10	75.26	UMB706, D03-D	EST, RAPD	5664.31	30.79	Badia
		***QRCsa-PD2-Y1-7H***	7H	3.99	19.23	− 49.10	Bmac0282b, EBmatc0016	SSR	2410.43	13.10	Kavir
	2019–2020	*QRCsa-PD2-Y2-2H*	2H	4.77	11.55	− 40.44	ISJ19-A	ISJ	1635.30	8.96	Kavir
		***QSCsa-PD2-Y2-3Ha***	3H	3.38	85.28	46.33	ISJ3-C, UMB310	ISJ, EST	2146.19	11.75	Badia
		*QRCsa-PD2-Y2-3Hb*	3H	3.85	124.44	− 34.80	Bmac0144k	SSR	1210.92	6.63	Kavir
		*QRCsa-PD2-Y2-4Ha*	4H	4.71	12.98	− 41.18	iPBS2415-1	iPBS	1696.16	9.29	Kavir
		*QSCsa-PD2-Y2-4Hb*	4H	3.02	33.81	31.03	ISJ9-B	ISJ	962.68	5.27	Badia
		***QSCsa-PD2-Y2-5H***	5H	3.15	87.10	48.23	UMB706, D03-D	EST, RAPD	2325.77	12.74	Badia
		***QRCsa-PD2-Y2-7H***	7H	5.88	18.49	− 48.76	Bmac0282b, EBmatc0016	SSR	2377.36	13.02	Kavir
	2020–2021	*QRCsa-PD2-Y3-2H*	2H	5.03	11.55	− 41.63	ISJ19-A	ISJ	1733.03	9.60	Kavir
		***QSCsa-PD2-Y3-3Ha***	3H	3.02	85.28	43.64	ISJ3-C, UMB310	ISJ, EST	1904.54	10.55	Badia
		*QRCsa-PD2-Y3-3Hb*	3H	3.96	124.44	− 35.34	Bmac0144k	SSR	1249.18	6.92	Kavir
		*QRCsa-PD2-Y3-4Ha*	4H	4.70	12.98	− 40.96	iPBS2415-1	iPBS	1677.66	9.29	Kavir
		*QSCsa-PD2-Y3-4Hb*	4H	3.55	33.81	33.54	ISJ9-B	ISJ	1125.13	6.23	Badia
		***QSCsa-PD2-Y3-5H***	5H	2.94	87.10	46.14	UMB706, D03-D	EST, RAPD	2128.96	11.79	Badia
		***QRCsa-PD2-Y3-7H***	7H	5.96	19.23	− 48.48	Bmac0282b, EBmatc0016	SSR	2349.83	13.01	Kavir
PD3^e^	2018–2019	***QRCsa-PD3-Y1-2H***	2H	3.49	15.49	− 49.69	SCoT7-C, ISJ17-B	SCoT, ISJ	2469.10	16.03	Kavir
		*QRCsa-PD3-Y1-3H*	3H	2.79	124.44	− 30.20	Bmac0144k	SSR	912.30	5.92	Kavir
		***QSCsa-PD3-Y1-5H***	5H	3.45	87.10	48.20	UMB706, D03-D	EST, RAPD	2323.46	15.08	Badia
		*QSCsa-PD3-Y1-7H*	7H	2.97	111.42	30.00	iPBS2257-A	iPBS	899.99	5.84	Badia
	2019–2020	***QRCsa-PD3-Y2-2H***	2H	3.65	15.49	− 49.55	SCoT7-C, ISJ17-B	SCoT, ISJ	2454.81	16.56	Kavir
		*QRCsa-PD3-Y2-3H*	3H	2.86	124.44	− 29.79	Bmac0144k	SSR	887.62	5.99	Kavir
		***QSCsa-PD3-Y2-5H***	5H	3.47	87.10	47.08	UMB706, D03-D	EST, RAPD	2216.74	14.96	Badia
		*QSCsa-PD3-Y2-7H*	7H	2.96	111.42	29.14	iPBS2257-A	iPBS	849.08	5.73	Badia
	2020–2021	***QSCsa-PD3-Y3-5H***	5H	4.69	87.10	62.73	UMB706, D03-D	EST, RAPD	3934.67	24.46	Badia

The major QTLs were marked with bold. The closet flanking marker to QTL was marked with an underline.

a Additive Effect, b Direction of Phenotypic Effect, c Early planting date, d Conventional planting date and e Delayed planting date.

### QTLs analysis in the conventional planting date or second planting date (PD2)

In the conventional planting date condition, the descriptive statistics (Supplementary Table 2) and Q–Q plot (Supplementary Fig. 3) showed that the AUDPC has normally distribute. Also, in this condition, the average phenotypic distribution of AUDPC revealed that the quantitative and continuous changes of AUDPC in SB disease (Supplementary Fig. 4). In addition, in studied population for AUDPC, transgressive segregation was observed in this condition.

In the conventional planting date condition, 16 QTLs were detected on chromosomes 2H, 3H, 4H, 5H and 7H (Table 1). Two minor QTLs including *QRCsa-PD2-Y2-2H* (LOD = 4.77, R^2^ = 8.96) and *QRCsa-PD2-Y3-2H* (LOD = 5.03, R^2^ = 9.60) were located on chromosome 2H with a marker distance of 11.55 cM in the stable region of ISJ19-A, and were in the Kavir direction (Supplementary Fig. 8, Table 1). Two QTLs including *QSCsa-PD2-Y2-3Ha* (LOD = 3.38) and *QSCsa-PD2-Y3-3Ha* (LOD = 3.02) were traced on chromosome 3H with a marker distance of 85.28 cM in the stable region of ISJ3-C-UMB310 (Supplementary Fig. 8, Table 1) and were in the Badia direction (Supplementary Fig. 8, Table 1). These QTLs were close to the flanking marker UMB310. *QSCsa-PD2-Y2-3Ha* and *QSCsa-PD2-Y3-3Ha* described 11.75% and 10.55% of phenotypic variation, respectively, and were major. Two QTLs including *QRCsa-PD2-Y2-3Hb* (LOD = 3.85, R^2^ = 6.63) and *QRCsa-PD2-Y3-3Hb* (LOD = 3.96, R^2^ = 6.92) were understood on chromosome 3H with a marker distance of 124.44 cM in the stable region of Bmac0144k (Supplementary Fig. 8, Table 1). These minor QTLs were in the Kavir direction and were exactly mapped on Bmac0144k (Table 1). Two QTLs including *QRCsa-PD2-Y2-4Ha* (LOD = 4.71, R^2^ = 9.29) and *QRCsa-PD2-Y3-4Ha* (LOD = 4.70, R^2^ = 9.29) were mapped on chromosome 4H with a marker distance of 12.98 cM in the stable region of iPBS2415-1 (Supplementary Fig. 8, Table 1). These minor QTLs were in the Kavir direction (Table 1). Two QTLs including *QSCsa-PD2-Y2-4Hb* (LOD = 3.02, R^2^ = 5.27) and *QSCsa-PD2-Y3-4Hb* (LOD = 3.55, R^2^ = 6.23) were found out on chromosome 4H with a marker distance of 33.81 cM in the stable region of ISJ9-B and were in the Badia direction (Supplementary Fig. 8, Table 1). *QSCsa-PD2-Y2-4Hb* had the lowest amount of genotypic variance (962.68) in this condition (Table 1). Three QTLs including *QSCsa-PD2-Y1-5H* (LOD = 4.98), *QSCsa-PD2-Y2-5H* (LOD = 3.15) and *QSCsa-PD2-Y3-5H* (LOD = 2.94) were discovered on chromosome 5H with a marker distance of 87.10 cM in the stable region of UMB706-D03-D (Supplementary Fig. 8, Table 1), also these major QTLs expressed 30.79%, 12.74% and 11.79% of the total genotypic variation, respectively. These QTLs were traced exactly between two flanking markers of UMB706 and D03-D. These QTLs have a positive additive effect; therefore, the selection was in the direction of removing the resistant alleles. In this condition, *QSCsa-PD2-Y1-5H* had the highest amount of genotypic variance (5664.31). Also, this QTL had the highest additive effect (75.26) in the Badia direction (Table 1). Three QTLs including *QRCsa-PD2-Y1-7H* (LOD = 3.99, marker distance = 19.23 cM), *QRCsa-PD2-Y2-7H* (LOD = 5.88, marker distance = 18.49 cM) and *QRCsa-PD2-Y3-7H* (LOD = 5.96, marker distance = 19.23 cM) were traced on chromosome 7H in the stable region of Bmac0282b-EBmatc0016 (Supplementary Fig. 8, Table 1). *QRCsa-PD2-Y1-7H* and *QRCsa-PD2-Y3-7H* were closer to the flanking marker of EBmatc0016; however, *QRCsa-PD2-Y2-7H* was closer to the flanking marker of Bmac0282b. In addition, *QRCsa-PD2-Y1-7H*, *QRCsa-PD2-Y2-7H* and *QRCsa-PD2-Y3-7H* had a coefficient of determination of 13.10, 13.02 and 13.01, respectively. These major QTLs had a negative additive effect and were in the Kavir direction. Furthermore, in this condition, *QRCsa-PD2-Y1-7H* had the highest additive effect (-49.10) in the aforementioned direction (Table 1).

### QTLs analysis in the delayed planting date or third planting date (PD3)

In the delayed planting date condition, the descriptive statistics (Supplementary Table 2) and Q–Q plot (Supplementary Fig. 5) revealed that the AUDPC has normally distribute. Also, in this condition, the average phenotypic distribution of AUDPC showed that the quantitative and continuous changes of AUDPC in SB disease (Supplementary Fig. 6). Moreover, transgressive segregation was detected on RILs population for AUDPC in this condition.

In the delayed planting date condition, nine QTLs were located on chromosomes 2H, 3H, 5H and 7H (Table 1). Two QTLs including *QRCsa-PD3-Y1-2H* (LOD = 3.49) and *QRCsa-PD3-Y2-2H* (LOD = 3.65) were realized on chromosome 2H with a marker distance of 15.49 cM in the stable region of SCoT7-C-ISJ17-B (Supplementary Fig. 9, Table 1). These QTLs were discovered closer to the flanking marker SCoT7-C. *QRCsa-PD3-Y1-2H* and *QRCsa-PD3-Y2-2H* were diagnosed as major with a coefficient of determination of 16.03 and 16.56, respectively. These QTLs have a negative additive effect; thus, the selection was in the direction of the presence of the resistant alleles. In this condition, *QRCsa-PD3-Y1-2H* had the highest additive effect (-49.69) in the Kavir direction (Table 1). Two QTLs including *QRCsa-PD3-Y1-3H* (LOD = 2.79, R^2^ = 5.92) and *QRCsa-PD3-Y2-3H* (LOD = 2.86, R^2^ = 5.99) were mapped on chromosome 3H with a marker distance of 124.44 cM in the stable region of Bmac0144k and were in the Badia direction (Supplementary Fig. 9, Table 1). Three QTLs including *QSCsa-PD3-Y1-5H* (LOD = 3.45), *QSCsa-PD3-Y2-5H* (LOD = 3.47) and *QSCsa-PD3-Y3-5H* (LOD = 4.69) were figured out on chromosome 5H with a marker distance of 87.10 cM in the stable region of UMB706-D03-D (Supplementary Fig. 9, Table 1). These QTLs were exactly between two flanking markers of UMB706 and D03-D. *QSCsa-PD3-Y1-5H*, *QSCsa-PD3-Y2-5H* and *QSCsa-PD3-Y3-5H* justified 15.08%, 14.96% and 24.46% of the total phenotypic variation, respectively; so, the selection of these major QTLs will be in the direction of removing the SB disease resistant alleles. *QSCsa-PD3-Y3-5H* had the highest amount of genotypic variance (3934.67) in this condition. Also, this QTL had the highest additive effect (62.73) in the Badia direction (Table 1). Two QTLs including *QSCsa-PD3-Y1-7H* (LOD = 2.97, R^2^ = 5.84) and *QSCsa-PD3-Y2-7H* (LOD = 2.96, R^2^ = 5.73) were discovered on chromosome 7H with a marker distance of 111.42 cM in the stable region of iPBS2257-A (Supplementary Fig. 9, Table 1). These minor QTLs were in the Badia direction. *QSCsa-PD3-Y2-7H* had the lowest amount of genotypic variance (849.08) in this condition (Table 1).

### Lines screening of resistance to SB and drought stress

**Figure 2:**
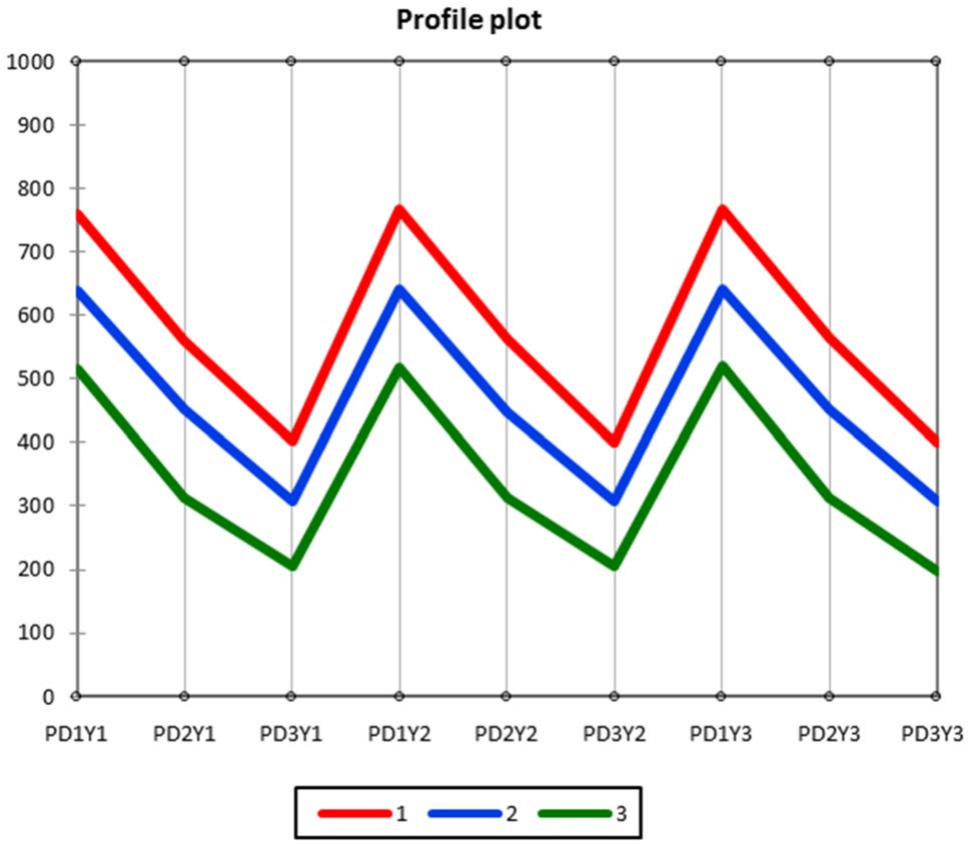
Profile plot for mean difference of AUDPC trait in spot blotch across clusters.

In different planting dates condition, the lines demonstrated a significant correlation (*P* ≤ 0.01) in terms of AUDPC in 2018/2019–2020/2021 crop seasons (Supplementary Table 3). This consistency indicates that the lines exhibited stability across environmental conditions and different years. Cluster analysis was employed to categorize the lines into three distinct groups. The first and third groups comprised 33, 27 and 43 lines, respectively (Supplementary Table 4). Within the first and third groups, the average AUDPC values for SB disease were 573.89, 467.05 and 347.11, respectively (Supplementary Table 5, Fig. 2). Accordingly, the first to third groups were designated as susceptible, semi-resistant and resistant lines, respectively. Following additional investigations, the lines belonging to the third cluster may be considered promising candidates in terms of purity, stability, and the presence of resistance genes against SB disease.

## Discussion

In the present study, drought stress was increased from 2018/2019 to 2020/2021 during the flowering stage. Because, the rainfall total in 2018/2019 to 2020/2021 crop seasons was 626.7, 376.3 and 246.1 mm, respectively (Supplementary Table 1). Moreover, the rainfall and relative humidity in February, April and March 2018/2019 were more than 2020/2021 (Supplementary Table 1). However, the temperature mean and evaporation mean in February, April and March 2018/2019 were less than 2020/2021 (Supplementary Table 1). Therefore, in February, April and March (2018/2019 to 2020/2021), the soil water potential (SWP) had a downward trend in the research farm of Gonbad Kavous University (Supplementary Table 6). As a result, in 2018/2019 to 2020/2021, the plants were exposed to different levels of osmotic potential on the same planting date. The results of this study are confirmed that drought stress was more intense at the end of the growing season, and as a result, to escape from the drought stress, early planting date is recommended in Gonbad Kavous region. Totally, it is necessary to provide the plant-water requirement (PWR) during the crop seasons. This is especially needful to provide the plant available water (PAW) during the stages sensitive to drought stress including germination, stem elongation, heading, flowering and graining. Thus, in tropical areas such as Gonbad Kavous region, four to five times irrigation is essential in barley fields during the growing stages.

In different planting dates, the transgressive segregation was detected on RILs population for AUDPC. Mutation, epistasis, over-dominance, complementary action of genes with additive effect and appearance of rare recessive allele in parents can all be considered as some of the most significant factors contributing to the transgressive segregation phenomenon^18,19^. In order to enhance the probability of transgressive segregated and to maintain the lines exhibiting transgressive segregation, breeding methods such as single seed descent (SSD) and recurrent breeding can be employed. The SSD method involves the removal of natural selection to maximize diversity in segregating generations. Moreover, the recurrent breeding method, by increasing the accumulation of desirable genes and disrupting genetic sequences, enhances the genetic foundation of the population and further increases the probability of transgressive segregation^20^.

The stable and closely linked QTLs of resistance to SB and drought stress including *QRCsa-2H* (ISJ19-A region), *QRCsa-2H* (SCoT7-C-ISJ17-B region), *QRCsa-3H* (Bmac0144k region), *QRCsa-4H* (iPBS2415-1 region) and *QRCsa-7H* (Bmac0282b-EBmatc0016 region) were mapped on chromosomes 2H, 3H, 4H and 7H (Fig. 1). To validate and confirm of the QTLs associated with resistance to SB, the studies were reviewed over the past twenty years. Overall, the QTLs of SB resistance were identified on chromosomes 1H^1,14,16,21–23^, 2H^1,21–23^, 3H^1,14,21–23^, 4H^1^, 5H^1,22^, 6H^1,23^ and 7H^1,14,16,22,23^ in barley. Based on this, *QRCsa-2H* (ISJ19-A region) was confirmed by Roy et al.^22^. Also, two QTLs including *QRCsa-3H* (Bmac0144k region) and *QRCsa-4H* (iPBS2415-1 region) were confirmed by Visioni et al.^1^. In addition, *QRCsa-7H* (Bmac0282b-EBmatc0016 region) was confirmed by Novakazi et al.^16^. However, *QRCsa-2H* (SCoT7-C-ISJ17-B region) was not identified in previous studies. Therefore, this QTL is likely novel. The important point is that the findings of all studies are not the same and the some QTLs were traced in different regions. Reasons for this include differences in parent types, population types, population size, marker types, sampling errors and experimental accuracy.

The bioinformatics studies showed the function of resistance QTLs to SB. The contig gene of *QRCsa-2H* (ISJ19-A region) produces carboxypeptidase enzyme (Table 2). The carboxypeptidase protein family uses serine or Zn (zinc) in the active site and plays a crucial role in response to stress, defense against pathogens and growth of the plant^24^. The contig gene of *QRCsa-2H* (SCoT7-C-ISJ17-B region) also produces the glycosyltransferase enzyme, which catalyzes the transfer of sugar (glycosyl) to inorganic phosphate or water, and is also active in polysaccharide, glycoprotein and glycolipid biosynthesis^25^. Examining the other contig gene of the *QRCsa-2H* showed that the enzyme produced in this genomic region is active in transferring certain substances from one side of the membrane to the other side of the membrane (Table 2). In this region of the genome of barley, QTLs of resistance to SFNB (Spot Form Net Blotch) were detected^12^. The contig gene product of *QRCsa-3H* (Bmac0144k region) is active as a transcription factor (Table 2). Furthermore, in this gene region, QTLs of resistance to SFNB in barley have been detected^12^. Additionally, the contig gene of *QRCsa-4H* (iPBS2415-1 region) was active as a kinase (Table 2). Finally, the bioinformatics studies, while investigating the function of a contig gene of *QRCsa-7H* (Bmac0282b-EBmatc0016 region), showed the activity of serine/threonine kinase protein as the product of this genomic region (Table 2). The MAPK (Mitogen-Activated Protein Kinase) cascade in plants plays an important role in various responses to biotic and abiotic stresses as well as in ROSs pathway and hormone signaling. As the last component of the cascade, MAPK showed the activity of serine/threonine protein as a product of this system^26^. MAPK is an important pathway that converts extracellular stimuli into intracellular responses and has three kinase components, including MAPK, MAPKK and MAPKKK^27^. Furthermore, the other contig gene in the *QRCsa-7H* region is involved in DNA binding in the AP2/ERF (APETALA2/Ethylene-Responsive Factor) gene family (Table 2). This gene family includes plant-specific transcription factors and plays a vital role in the developmental processes, resistance to biotic and abiotic stresses, and response to plant hormones^28^. In this region of chromosome 7H, QTLs of resistance to SFNB disease were detected^12^.

**Table 2:** The genes, protein names and molecular function were associated with stable and closely linked QTLs of resistance against spot blotch and drought stress in barley (*Hordeum vulgare* L). Available at: https://www.uniprot.org [Accessed 14 August 2022]^48^.

QTL	Marker	Gene name	Protein name	Molecular function	Length (AA)
*QRCsa-2H*	ISJ19-A	HORVU.MOREX.r3.4HG0345700	Carboxypeptidase	Response to stress	472
*QRCsa-2H*	SCoT7-C	HORVU.MOREX.r3.2HG0107160	Glycosyltransferase (GTFs)	Transferase activity	467
	ISJ17-B	HORVU.MOREX.r3.2HG0155590	Predicted protein	Transmembrane transporter activity	577
*QRCsa-3H*	Bmac0144k	HORVU.MOREX.r3.5HG0433910	Predicted protein	DNA-binding transcription factor activity	355
*QRCsa-4H*	iPBS2415-1	HORVU.MOREX.r3.6HG0538650	Protein kinase domain-containing protein	Protein kinase activity	613
*QRCsa-7H*	Bmac0282b	HORVU.MOREX.r3.1HG0085350	Protein kinase domain-containing protein	Protein serine/threonine kinase activity	348
	EBmatc0016	HORVU.MOREX.r3.7HG0662720	AP2/ERF domain-containing protein	DNA binding	198

The QTLs of resistance to disease are used in advanced plant breeding such as gene pyramiding^29^. In addition, by using map-based cloning, it is possible to clone the QTLs of resistance to SB. Also, the flanking markers of resistance QTLs can simultaneously positive screen cultivars for resistance to SB disease and drought stresses during the reproductive stage; therefore, after confirming the QTLs by fine mapping method, they can be used in MAS projects^30^. For instance, in a study using BSA (Bulked Segregant Analysis) and fine mapping, the exact location of a major *Rbs7* gene on chromosome 6HS in the M13.37-M13.06 regions (physical distance of 304 kb) was detected^30^. Accordingly, in the early or conventional planting dates, SB-resistant cultivars are screened using Bmac0282b and EBmatc0016 markers. Moreover, in the early or delayed planting dates, SCoT7-C and ISJ17-B markers are recommended for positive screening of SB-resistant cultivars. Also, in the conventional planting date, two flanking markers including ISJ19-A and iPBS2415-1 are suggested for the positive screening of SB-resistant cultivars. Finally, in different planting dates, a flanking marker named Bmac0144k can be used for screening of SB-resistant cultivars.

The stable and closely linked QTLs of susceptibility to SB and drought stress including *QSCsa-3H* (ISJ3-C-UMB310 region), *QSCsa-4H* (ISJ9-B region), *QSCsa-5H* (UMB706-D03-D region) and *QSCsa-7H* (iPBS2257-A region) were detected on chromosomes 3H, 4H, 5H and 7H (Fig. 1). The QTLs validation were done by evaluating the findings of other studies over the past twenty years. However, only a study has been conducted on QTLs of susceptibility to SB in barley. Leng et al.^17^ showed that the QTLs of SB susceptibility were traced on chromosomes 1H (flanking markers: 1H_20693748-1H_486185365) and 7H (flanking markers: 7H_18088775-7H_45702908) using QTL interval mapping technique and SNPs^17^. As a result, four QTLs of susceptibility to SB including *QSCsa-3H* (ISJ3-C-UMB310 region), *QSCsa-4H* (ISJ9-B region), *QSCsa-5H* (UMB706-D03-D region) and *QSCsa-7H* (iPBS2257-A region) were not identified in other studies. Thus, the last four QTLs are likely novel. It is noteworthy that some cultivars susceptible to disease have a high genetic potential for functioning, and their negative traits can be bred by editing the disease-susceptible regions, while the agronomical values of the cultivar will be preserved. For this purpose, CRISPR/Cas9 technology can be utilized^31^. Also, to remove cultivars susceptible to SB disease from germplasm collection in MAS projects, negative screening of susceptible cultivars can be used^12^. Based on this, in the conventional planting date condition, UMB310, ISJ3-C and ISJ9-B markers can be utilized for the negative screening of SB-susceptible cultivars. Also, in the delayed planting date, iPBS2257-A marker is recommended for the negative screening of SB-susceptible cultivars. Finally, in different planting dates, markers UMB706 and D03-D are available for negative screening of SB-susceptible cultivars. The QTLs of susceptibility to SFNB disease were traced in the recent region^12^.

In the current study, some QTLs of SB resistance/susceptibility were unstable in different years. Because, in 2018/2019–2020/2021, Gonbad Kavous region had rainy, conventional and dry conditions, respectively (Supplementary Table 1). Moreover, the rainfall distribution were different during the three years, which is not showed in Supplementary Table 1. In addition, the QTLs had epistasis on each other. We recommend that the QTLs identified in present study are first evaluated in different environments, regions and parents with population types, large population size and more markers. After confirmation, the QTLs can be used in fine mapping and screening projects (MAS).

## Conclusions

This study is the first report of common resistance to SB and drought stress in barley. A total of 43 lines had resistance to SB and drought stress. These promising lines can be used to produce of resistant variety. These lines have high genetic resistance potential, and therefore after additional studies, recommended for use in variety production. Overall, five stable and closely linked QTLs of resistance to SB and drought stress including *QRCsa-2H* (ISJ19-A region), *QRCsa-2H* (SCoT7-C-ISJ17-B region), *QRCsa-3H* (Bmac0144k region), *QRCsa-4H* (iPBS2415-1 region) and *QRCsa-7H* (Bmac0282b-EBmatc0016 region) were located on chromosomes 2H, 3H, 4H and 7H. The bioinformatics studies were determined the molecular function of resistance QTLs to SB and drought stress. Based on this, *QRCsa-2H* (11.55 cM), *QRCsa-2H* (15.49 cM), *QRCsa-3H* (124.44 cM), *QRCsa-4H* (12.98 cM) and *QRCsa-7H* (18.49 cM) were involved in the carboxypeptidase, Glycosyltransferase (transferase activity), DNA-binding transcription factors, protein kinase pathway and AP2/ERF family, respectively. Moreover, four stable and closely linked QTLs of susceptibility to SB and drought stress including *QSCsa-3H* (ISJ3-C-UMB310 region), *QSCsa-4H* (ISJ9-B region), *QSCsa-5H* (UMB706-D03-D region) and *QSCsa-7H* (iPBS2257-A region) were mapped on chromosomes 3H, 4H, 5H and 7H, and were likely novel.

The flanking markers are used in MAS projects to screen cultivars. Accordingly, the flanking markers of resistance regions to SB and drought stress including ISJ19-A, SCoT7-C, ISJ17-B, Bmac0144k, iPBS2415-1, Bmac0282b and EBmatc0016 can be used in the positive screening of resistant cultivars. However, the flanking markers of susceptibility regions to SB and drought stress including ISJ3-C, UMB310, ISJ9-B, UMB706, D03-D and iPBS2257-A can be utilized in the negative screening of susceptible cultivars in MAS projects.

All in all, the basis of cellular, functional and molecular mechanisms of resistance to biotic and abiotic stress are the same in plants. In addition, the stable QTLs of resistance to different diseases in a plant species is similar. Therefore, a QTL of resistance against various diseases has a high genetic potential in inducing durable resistance against various stresses. The some high-yielding cultivars are susceptible to SB in Gonbad Kavous region of Iran. Hence, it is necessary to transfer such QTLs of resistance to these cultivars.

## Methods

### Plant materials and experiment conditions

In order to trace the QTLs associated with resistance/susceptibility trait to SB disease and drought stress in barley, an experiment was conducted with 103 F8 lines resulting from the cross Badia (as female) and Kavir (as male) cultivars. This experiment was conducted as an α-lattice design with three replications in the research farm of Gonbad Kavous University (37o 17´ N, 55o 18´ E, altitude 38 m) in 2018/2019 to 2020/2021. The population was developed to present the plant genetic materials under the Gonbad Kavous University’s license.

All the methods were performed in accordance with relevant guidelines and regulations. Badia (six-rowed, susceptibility to SB and drought) and Kavir (six-rowed, resistance to SB and drought) cultivars are licensed by ICARDA (International Center for Agricultural Research in the Dry Areas) and SPII (Seed and Plant Improvement Institute), respectively. The Badia cultivar is susceptible to powdery mildew, lodging, drought and salinity. However, the Kavir cultivar is semi-resistant to powdery mildew and lodging, is semi-susceptible to grain shattering, is resistant to drought and has early maturity^12^. Using meteorological data (Supplementary Table 1), the early (October 7), conventional (December 13) and delayed (January 19) planting dates^32^ were adjusted in such a way that the flowering stages of the plant coincide with different osmotic potentials (Supplementary Table 6). The flowering stage in the early, conventional and delayed planting dates coincided with late February, early April and early May, respectively. In addition, the experiment was conducted as dryland farming to prevent the possibility of reducing drought effects with irrigation. Each cultivar or line was cultivated on a two-meter-long row and the rows were 20 cm apart. Seeds were planted with a density of 270 plants/m^2^. All crop care activities were performed according to international protocols.

### Inoculum preparation and disease measurement

First, *Cochliobolus sativus* Gonbad isolate was obtained from IRIPP (Iranian Research Institute of Plant Protection). The single-spore isolate was transferred to V8-PDA medium and kept in the dark/light (8/16 h) at 25 °C. Conidia of the pathogen were harvested by adding a few milliliters of sterile distilled water and scraping the surface of the culture medium using a sterile specula followed by filtration with double layer of cheese cloth to remove the fungal mycelium. The conidial concentration was adjusted to 8000 conidia/ml by adding sterile distilled water supplemented with 100 µl/l of Tween 20 to promote a uniform distribution and adhesion of conidia to barley leaves^22^. In the greenhouse, seedlings of susceptible cultivar “Afzal” was inoculated at the Zadoks growth stage^33^ (GS) 13 with spore suspension using hand held sprayers (0.4 ml/seedling) till run off^1^. The inoculated seedlings were incubated for 18–24 h in the dark at 25–27 °C and high humidity (saturation). Then, they were transferred to a growth chamber with humidity of 75% and a photoperiod (16-h light/8-h dark) at 25–27 °C. Finally, the biomass was harvested and divided into small pieces during the adult stage.

The susceptible cultivar “Afzal” was planted along the border and within the rows of accession as disease spreader, to support *C. sativus* infection. Pieces of infected green plant material (with a rate of 50 gr of straw per meter of planting row) was used as inoculum when the spreaders were at the Zadoks GS^33^ 24 (tillering)^34^, and were distributed between the plants of the susceptible cultivar and in the middle of the planting row of the accessions to inoculate the field. After inoculation, the field was flooded to ensure infection and enhance development of SB disease. Mist irrigation system was also applied to create saturated moisture in the field^16^.

Disease assessment was done using the double-digit scale (00–99), where the first digit refers to the relative height of the disease progress in canopy, and the second digit indicates disease severity based on the percentage of leaf necrosis^35^. Disease was evaluated five times at seven-day intervals during the Zadoks GSs^33^ 50–80. Then, AUDPC was calculated using the following Eq. ^36^:

$${\text{AUDPC}} = \mathop \sum \limits_{i = 1}^{n} \frac{{\left( {SB_{i + 1} + SB_{i} } \right)\left( {t_{i + 1} - t_{i} } \right)}}{2}$$

In this equation, *n* is the number of observations, *SBi* is the severity of SB disease on the *i-*th day, and *t* is the number of days after cultivation.

### Evaluation of phenotypic data

The descriptive statistics of the phenotypic data were calculated and using Pearson test the normality of the data was checked in terms of skewness and kurtosis. Furthermore, to ensure the normality of the data, phenotypic distribution figures and Q–Q plot were also used. Then, in order to ensure the stable reaction of the lines in different environmental conditions, using Pearson method, the average correlation of the treatments was calculated. Finally, to separate lines resistant and susceptible to drought stress, cluster analysis according to the Euclidean distance and WARD (minimum variance) method were used, and also the cutting location was determined using discriminant analysis based on 1000 times bootstrap. In addition, the accuracy of the cluster results was confirmed with cophenetic correlation coefficient. All the aforementioned calculations were done with R version 4.2.2 software and the figures were arranged with XLSTAT software.

### Evaluation of genotypic data

First, leaf samples were taken from the parents and the population of RILs. Then, using CTAB (cetyl trimethyl ammonium bromide) method, genomic DNA was extracted^37^. The quality and quantity of DNA samples were controlled by horizontal electrophoresis (0.8% agarose gel) and spectrophotometry, respectively. PCR (Polymerase Chain Reaction) with a thermocycler device (Bio-Rad USA) was used to amplify genomic DNA. In order to increase the accuracy and to prepare the specific amplicon, thermal programs and time periods were used by TD PCR (touchdown PCR) method^38^. The PCR reaction solution for SSR (single sequence repeat or microsatellite) markers included 2.5 µl of DNA, 0.48 µl of MgCl_2_ (50 mM), 0.6 µl of dNTP (10 mM), 0.75 µl of forward primer (10 pmol), 0.75 µl of reverse primer (10 pmol), 1 µl of PCR buffer, 0.12 µl of *Taq* DNA polymerase (5 U/µl) and 3.8 µl of sterile water; and the volume of the reaction solution was brought to 10 µl. For dominant markers, the reaction solution was similarly prepared, however, 1.5 µl of primer were used. Then, to separate the bands, vertical electrophoresis was used and to do so, the PCR product was loaded on the 6% polyacrylamide gel. For coloring the bands, the silver staining method was used^39^. Then, the molecular data were recorded and the genetic matrix was arranged. Finally, the expected Mendelian ratio (1:1) for the population genotype was tested using χ^2^ test with SAS software.

### Genetic linkage map

Polymorphism between parents was investigated using co-dominant and dominant markers including SSR, ISSR (Inter Simple Sequence Repeat), CBDP (CAAT Box-Derived Polymorphism), EST (Expressed Sequence Tag), SCoT (Start Codon Target), TE (Transposable Element), RAPD (Random Amplified Polymorphism DNA), ISJ (Intron–exon Splice Junctions), IRAP (Inter-Retrotransposon Amplified Polymorphism), iPBS (inter Primer Bindings Site), iPBS-iPBS combined and ISSR-iPBS combined. Then, using polymorphic markers, polymorphism was determined among F_8_ individuals. Co-dominant and dominant markers were used to detect linkage groups and saturation linkage map, respectively. Co-dominant markers were selected in such a way that there are at least a few markers on both short and long arms in all barley chromosomes^40^. In addition, the dominant markers were selected based on the previous research in terms of PIC (polymorphic information content), gene diversity (heterozygosity) and allele number. The linkage map was prepared with Map Manager QTX17 software^41^ and the distance between the markers was determined in cM unit using Kosambi function^42^.

### QTL analysis

Multi-QTL mapping was the advanced method for QTL mapping^43^. However, it was difficult to identify minor and closely linked QTLs^43^. The reasons include the large experimental error and limited number of lines^43^. Therefore, a new method called genome-wide composite interval mapping (GCIM) was suggested^44,45^. Overall, GCIM has high accuracy in estimating QTL parameters, high power in QTL identification and low false positive rate as compared with the widely-used methods^43,45^. Therefore, in this study, the closely linked QTLs were identified using GCIM-random with a two cM genome walking. This method was based on the FASTmrEMMA (fast multi-locus random-SNP-effect efficient mixed model association) algorithm^46^. In addition, the restricted maximum likelihood or maximum likelihood can be used to estimate the parameters in GCIM-random^45^. The polygenic additive variance was calculated via mixed model framework of GWAS^45^. The LOD (logarithm of the odds) threshold was selected using permutation test with 1000 replications. QTLs.gCIMapping.GUI v2.0 package in R software was used to conduct this method^43^. In addition, coefficient of determination (R^2^) was used to detect for minor and major QTLs, and then, QTLs with a R^2^ higher than 10% were considered as major ones^12^. Furthermore, the naming of QTLs was written according to international methods from left to right, Q (abbreviation for QTL), R (abbreviation for resistant), S (abbreviation for susceptible), different planting dates (PD1, PD2 and PD3 abbreviations for early, conventional and delayed planting date, respectively), Y (abbreviation for year), and chromosome number.

### Identifying the function of resistance genes

Finally, the genes and proteins were associated with stable and closely linked QTLs of resistance against SB and drought stress, were detected. For this purpose, the important genes were identified using Morex reference genome. Then, the contig genes were traced using sequence of the flanking markers^2,47^ and the contig gene’s function were identified^48^.

### Supplementary Information

Supplementary Information.

## Data Availability

The data that support the results of this study are available from the corresponding author upon request.

## References

[1] Visioni A (2020). Genome wide association mapping of spot blotch resistance at seedling and adult plant stages in barley. Front. Plant Sci..

[2] Ensemble Plants. http://plants.ensembl.org (2022).

[3] Guo H (2019). Virulence and molecular diversity in the *Cochliobolus sativus* population causing barley spot blotch in China. Plant Dis..

[4] Oliver, R. Integrated disease management of wheat and barley (Burleigh Dodds Science Publishing, 2018).

[5] Gusta LV, Fowler DB (1976). Effects of temperature on deharding and reharding of winter cereals. Can. J. Plant Sci..

[6] Sallam A, Alqudah AM, Dawood MFA, Baenziger PS, Börner A (2019). Drought stress tolerance in wheat and barley: Advances in physiology, breeding and genetics research. Int. J. Mol. Sci..

[7] Tanaka R, Nakano H (2019). Barley yield response to nitrogen application under different weather conditions. Sci. Rep..

[8] Kazerani B (2019). Evaluation of proline content and enzymatic defense mechanism in response to drought stress in rice. Iran. J. Plant Physiol..

[9] Kazerani B (2019). Study of antioxidant defense genes expression in rice (Oryza sativa L.) cultivars in response to drought stress. Iran. J. Genet. Plant Breed..

[10] Kazerani B, Navabpour S (2019). Induced genes expression pattern in response to drought stress at seedling stage of wheat. J. Plant Physiol. Breed..

[11] Kazerani B (2019). Grouping of rice mutant lines based on morphological and agronomical traits under different moisture conditions using multivariate statistical methods. J. Plant Physiol. Breed..

[12] Sabouri H, Taliei F, Kazerani B, Ghasemi S, Katouzi M (2023). Identification of novel and stable genomic regions associated with barley resistance to spot form net blotch disease under different temperature conditions during the reproductive stage. Plant Pathol..

[13] Sabouri H (2022). Association analysis of yellow rust, fusarium head blight, tan spot, powdery mildew, and brown rust horizontal resistance genes in wheat. Physiol. Mol. Plant Pathol..

[14] Zhou H, Steffenson B (2013). Genome-wide association mapping reveals genetic architecture of durable spot blotch resistance in US barley breeding germplasm. Mol. Breed..

[15] Leng Y (2018). The gene conferring susceptibility to spot blotch caused by *Cochliobolus sativus* is located at the Mla locus in barley cultivar Bowman. Theor. Appl. Genet..

[16] Novakazi F (2020). Genome-wide association studies in a barley (*Hordeum vulgare*) diversity set reveal a limited number of loci for resistance to spot blotch (*Bipolaris sorokiniana*). Plant Breed..

[17] Leng Y (2020). Molecular mapping of loci conferring susceptibility to spot blotch and resistance to powdery mildew in barley using the sequencing-based genotyping approach. Phytopathology.

[18] Mackay IJ, Cockram J, Howell P, Powell W (2021). Understanding the classics: the unifying concepts of transgressive segregation, inbreeding depression and heterosis and their central relevance for crop breeding. Plant Biotechnol. J..

[19] Rieseberg LH, Archer MA, Wayne RK (1999). Transgressive segregation, adaption and speciation. Hered..

[20] Kuczynska A, Surma M, Adamski T (2007). Methods to predict transgressive segregation in barley and other self-pollinated crops. J. Appl. Genet..

[21] Bykova IV, Lashina NM, Efimov VM, Afanasenko OS, Khlestkina EK (2017). Identification of 50 K Illumina-chip SNPs associated with resistance to spot blotch in barley. BMC Plant Biol..

[22] Roy JK (2010). Association mapping of spot blotch resistance in wild barley. Mol. Breed..

[23] Wang R, Leng Y, Ali S, Wang M, Zhoung S (2017). Genome-wide association mapping of spot blotch resistance to three different pathotypes of *Cochliobolus sativus* in the USDA barley core collection. Mol. Breed..

[24] Xu X (2021). Genome-wide analysis of the serine carboxypeptidase-like protein family in *Triticum aestivum* reveals *TaSCPL184-6D* is involved in abiotic stress response. BMC Genom..

[25] Zabotina OA, Zhang N, Weerts R (2021). Polysaccharide biosynthesis: Glycosyltransferases and their complexes. Front. Plant Sci..

[26] Goyal RK (2018). Analysis of MAPK and MAPKK gene families in wheat and related *Triticeae* species. BMC Genom..

[27] Chen X (2021). The role of the MAP kinase-kinase protein StMKK1 in potato immunity to different pathogens. Hortic. Res..

[28] Guo B, Wei Y, Xu R, Lin S, Luan H, Lv C, Zhang X, Song X, Xu R (2016). Genome-wide analysis of APETALA2/ethylene-responsive factor (AP2/ERF) gene family in barley (Hordeum vulgare L.). PLoS One..

[29] Ramalingam J (2020). Gene pyramiding for achieving enhanced resistance to bacterial blight, blast, and sheath blight diseases in rice. Front. Plant Sci..

[30] Wang R, Leng Y, Zhao M, Zhong S (2019). Fine mapping of a dominant gene conferring resistance to spot blotch caused by a new pathotype of *Bipolaris sorokiniana* in barley. Theor. Appl. Genet..

[31] Wang JY, Doudna JA (2023). CRISPR technology: A decade of genome editing is only the beginning. Science.

[32] Wiegmann M (2019). Barley yield formation under abiotic stress depends on the interplay between flowering time genes and environmental cues. Sci. Rep..

[33] Zadoks JC, Chang TT, Konzak CF (1974). A decimal code for the growth stages of cereals. Weed Res..

[34] Bilgic H, Steffenson BJ, Hayes PM (2006). Molecular mapping of loci conferring resistance to different pathotypes of the spot blotch pathogen in barley. Phytopathology.

[35] Nagarajan S, Kumar J, Duveiller E, Dubin HJ, Reeves J, McNab A (1998). Foliar blights of wheat in India. Helminthosporium Blights of Wheat: Spot Blotch and Tan Spot.

[36] Jeger MJ, Viljanen-Rollinson SLH (2001). The use of the area under the disease-progress curve (AUDPC) to assess quantitative disease resistance in crop cultivars. Theor. Appl. Genet..

[37] Stein N, Herren G, Keller B (2001). A new DNA extraction method for high-throughput marker analysis in a large-genome species such as *Triticum aestivum*. Plant Breed..

[38] Korbie D, Mattick J (2008). Touchdown PCR for increased specificity and sensitivity in PCR amplification. Nat. Protoc..

[39] Liang Q (2014). A rapid and effective method for silver staining of PCR products separated in polyacrylamide gels. Electrophor..

[40] A Database for *Triticeae* and *Avena*. http://www.graingenes.org (2022).

[41] Manly KF, Cudmore RH, Meer JM (2001). Map Manager QTX, cross-platform software for genetic mapping. Mamm. Genome.

[42] Kosambi DD (1943). The estimation of map distances from recombination values. Ann. Hum. Genet..

[43] Zhang YW, Wen YJ, Dunwell JM, Zhang YM (2020). QTL.gCIMapping.GUI v2.0: An R software for detecting small-effect and linked QTLs for quantitative traits in bi-parental segregation populations. Comput. Struct. Biotechnol. J..

[44] Wang SB (2016). Mapping small-effect and linked quantitative trait loci for complex traits in backcross or DH populations via a multi-locus GWAS methodology. Sci. Rep..

[45] Wen YJ (2019). An efficient multi-locus mixed model framework for the detection of small and linked QTLs in F_2_. Brief. Bioinform..

[46] Wen YJ (2018). Methodological implementation of mixed liner models in multi-locus genome-wide association studies. Brief. Bioinform..

[47] Bolser, D., Staines, D. M., Pritchard, E. & Kersey, P. Ensembl plants: Integrating Tools for Visualizing, Mining, and Analyzing Plant Genomics Data in *Plant Bioinformatics. Methods in Molecular Biology* (ed. Edwards, D.) 400p. (Humana Press, New York, NY, 2016). 10.1007/978-1-4939-3167-5_6 26519403

[48] UniProt (Universal Protein Resource) consortium database. https://www.uniprot.org (2022).

