# Supplementary material for: Finding stable and closely linked QTLs against spot blotch in different planting dates during the adult stage in barley
Source: Sci Rep. 2024 Jan 8;14:818. doi: 10.1038/s41598-024-51358-3 (PMC10774436; doi:10.1038/s41598-024-51358-3)
Supplement: Supplementary file 1 — Supplementary Information. [file 41598_2024_51358_MOESM1_ESM.docx]

**Supplementary Information**

**Manuscript title**

Finding stable and closely linked QTLs against spot blotch in different planting dates during the adult stage in barley

**Authors list:**

Fakhtak Taliei^a*^, Hossein Sabouri^a^, Borzo Kazerani^b^, Shahram Ghasemi^a^

1. Department of Plant Production, College of Agriculture Science and Natural Resources, Gonbad Kavous University, Gonbad Kavous, Iran.
2. Department of Plant Breeding and Biotechnology, Faculty of Plant Production, Gorgan University of Agricultural Science and Natural Resources, Gorgan, Iran.

^*^Corresponding author, E-mail address: taliei@gonbad.ac.ir

**Content**

| Supplementary Table 1 | …………………………………………………………………………………… | 2 |
| --- | --- | --- |
| Supplementary Table 2 | …………………………………………………………………………………… | 2 |
| Supplementary Table 3 | …………………………………………………………………………………… | 3 |
| Supplementary Table 4 | …………………………………………………………………………………… | 3 |
| Supplementary Table 5 | …………………………………………………………………………………… | 3 |
| Supplementary Table 6 | …………………………………………………………………………………… | 3 |
| Supplementary Figure 1 | …………………………………………………………………………………… | 4 |
| Supplementary Figure 2 | …………………………………………………………………………………… | 5 |
| Supplementary Figure 3 | …………………………………………………………………………………… | 6 |
| Supplementary Figure 4 | …………………………………………………………………………………… | 7 |
| Supplementary Figure 5 | …………………………………………………………………………………… | 8 |
| Supplementary Figure 6 | …………………………………………………………………………………… | 9 |
| Supplementary Figure 7 | …………………………………………………………………………………… | 10 |
| Supplementary Figure 8 | …………………………………………………………………………………… | 11 |
| Supplementary Figure 9 | …………………………………………………………………………………… | 12 |

**Supplementary Table 1.** Meteorological data of crop years 2018/2019, 2019/2020 and 2020/2021 in Gonbad Kavous region.

| Jun. | May. | Apr. | Mar. | Feb. | Jan. | Dec. | Nov. | Oct. | Index | Year |
| --- | --- | --- | --- | --- | --- | --- | --- | --- | --- | --- |
| 6.3 | 41.5 | 51.2 | 167.1 | 152.3 | 81.2 | 63.7 | 30.6 | 32.8 | Rainfall Total (mm) | 2018-2019 |
| 55 | 67 | 80 | 70.5 | 78 | 73 | 78 | 67 | 61 | Relative Humidity (%) |  |
| 43.8 | 38 | 33.5 | 29.7 | 25.2 | 26.3 | 26.1 | 39.7 | 36.1 | Absolute maximum temperature (^O^C) |  |
| 36.3 | 28.2 | 20.7 | 19.4 | 15.2 | 15.9 | 17 | 22.3 | 28.8 | Mean maximum temperature (^O^C) |  |
| 28.8 | 20.8 | 15.5 | 12.3 | 9.2 | 10.4 | 12.3 | 16 | 21.3 | Temperature Mean (^O^C) |  |
| 20.1 | 13.5 | 10.3 | 5.2 | 4.3 | 4.9 | 7.7 | 9.7 | 13.9 | Mean minimum temperature (^O^C) |  |
| 12.4 | 3.6 | 3.2 | -2 | -1.2 | -1 | 3.4 | 4.6 | 8 | Absolute minimum temperature (^O^C) |  |
| 236.1 | 134.2 | 62.8 | 61.9 | 37.2 | 40.6 | 45.1 | 66.4 | 117.3 | Evaporation Mean (mm) |  |
| 0 | 0 | 0 | 1 | 3 | 1 | 0 | 0 | 0 | Number of days with negative temperature |  |
| 2.4 | 40.6 | 93.2 | 65.9 | 68.4 | 16.4 | 11.9 | 54.6 | 22.9 | Rainfall Total (mm) | 2019-2020 |
| 48 | 73 | 81 | 77 | 64 | 66 | 74 | 72 | 61 | Relative Humidity (%) |  |
| 46.9 | 36.1 | 30.3 | 26.9 | 31.7 | 28.8 | 16.8 | 30.9 | 36 | Absolute maximum temperature (^O^C) |  |
| 36.4 | 26.3 | 19.3 | 19 | 17 | 17.1 | 18.1 | 21.6 | 30.3 | Mean maximum temperature (^O^C) |  |
| 27.6 | 19.6 | 13.7 | 12.4 | 9.9 | 10.4 | 11.8 | 15.1 | 22.3 | Temperature Mean (^O^C) |  |
| 18.7 | 12.9 | 8.2 | 5.8 | 2.8 | 3.8 | 5.4 | 8.6 | 14.4 | Mean minimum temperature (^O^C) |  |
| 12.8 | 8.4 | 0 | 0.4 | -2.1 | -0.5 | -0.5 | 1.7 | 7.7 | Absolute minimum temperature (^O^C) |  |
| 225.4 | 99.5 | 51.4 | 46.1 | 51.9 | 43 | 38 | 48.3 | 113.2 | Evaporation Mean (mm) |  |
| 0 | 0 | 1 | 0 | 6 | 1 | 1 | 0 | 0 | Number of days with negative temperature |  |
| 12.2 | 20 | 16.6 | 62.2 | 24.5 | 31.8 | 31.9 | 16.5 | 30.4 | Rainfall Total (mm) | 2020-2021 |
| 50 | 62 | 67 | 73 | 71 | 72 | 78 | 66 | 57 | Relative Humidity (%) |  |
| 46.5 | 43.8 | 34.3 | 35.9 | 30.1 | 29.6 | 20 | 37.2 | 32.4 | Absolute maximum temperature (^O^C) |  |
| 36.3 | 29.8 | 23.6 | 15.5 | 17.6 | 14.7 | 13.6 | 23 | 27.6 | Mean maximum temperature (^O^C) |  |
| 28.2 | 22.3 | 16.6 | 9.1 | 10.2 | 8.3 | 8.9 | 16.5 | 20.3 | Temperature Mean (^O^C) |  |
| 20.1 | 14.9 | 9.5 | 2.7 | 2.9 | 1.9 | 4.2 | 10.1 | 13 | Mean minimum temperature (^O^C) |  |
| 14.8 | 12.2 | 2.6 | -5.7 | -3.9 | -3.1 | -4 | 1.5 | 6.9 | Absolute minimum temperature (^O^C) |  |
| 225.9 | 149 | 99.1 | 43.6 | 47.1 | 40.2 | 24.8 | 70.1 | 112.4 | Evaporation Mean (mm) |  |
| 0 | 0 | 0 | 11 | 6 | 9 | 4 | 0 | 0 | Number of days with negative temperature |  |

**Supplementary Table 2.** Descriptive statistics for estimated AUDPC in spot blotch disease barley.

| **Statistic** | **PD1Y1** | **PD1Y2** | **PD1Y3** | **PD2Y1** | **PD2Y2** | **PD2Y3** | **PD3Y1** | **PD3Y2** | **PD3Y3** |
| --- | --- | --- | --- | --- | --- | --- | --- | --- | --- |
| Minimum | 375.93 | 375.93 | 380.45 | 133.95 | 132.65 | 134.28 | 102.31 | 103.70 | 35.62 |
| Kavir | 413.74 | 429.73 | 431.12 | 177.93 | 168.63 | 171.38 | 160.39 | 153.12 | 83.11 |
| Badia | 849.32 | 838.55 | 822.17 | 658.91 | 646.74 | 651.82 | 522.17 | 509.42 | 501.08 |
| Maximum | 933.33 | 935.24 | 934.28 | 732.36 | 734.57 | 735.39 | 596.30 | 586.23 | 574.26 |
| Mean | 625.79 | 630.16 | 630.93 | 428.07 | 427.32 | 430.80 | 294.81 | 294.03 | 292.00 |
| Variance | 13883.34 | 13813.57 | 13750.33 | 13988.65 | 14156.93 | 14252.24 | 9440.75 | 9011.26 | 10070.06 |
| Standard deviation | 117.83 | 117.53 | 117.26 | 118.27 | 118.98 | 119.38 | 97.16 | 94.93 | 100.35 |
| Skewness (Pearson) | 0.116 | 0.116 | 0.093 | 0.008 | 0.025 | 0.005 | 0.250 | 0.168 | 0.043 |
| Kurtosis (Pearson) | -0.404 | -0.460 | -0.491 | -0.488 | -0.485 | -0.475 | 0.034 | -0.088 | 0.005 |

PD1, PD2 and PD3 are early, conventional and delayed planting dates, respectively. Y1, Y2 and Y3 are 2018/2019, 2019/2020 and 2020/2021, respectively

**Supplementary Table 3.** Pearson correlation coefficients for mean AUDPC trait of spot blotch in different planting dates.

|  | PD1Y1 | PD1Y2 | PD1Y3 | PD2Y1 | PD2Y2 | PD2Y3 | PD3Y1 | PD3Y2 | PD3Y3 |
| --- | --- | --- | --- | --- | --- | --- | --- | --- | --- |
| PD1Y1 | 1 |  |  |  |  |  |  |  |  |
| PD1Y2 | **0.998** | 1 |  |  |  |  |  |  |  |
| PD1Y3 | **0.998** | **0.999** | 1 |  |  |  |  |  |  |
| PD2Y1 | **0.992** | **0.993** | **0.994** | 1 |  |  |  |  |  |
| PD2Y2 | **0.993** | **0.994** | **0.995** | **0.999** | 1 |  |  |  |  |
| PD2Y3 | **0.992** | **0.993** | **0.994** | **0.999** | **0.999** | 1 |  |  |  |
| PD3Y1 | **0.994** | **0.993** | **0.993** | **0.990** | **0.991** | **0.989** | 1 |  |  |
| PD3Y2 | **0.994** | **0.993** | **0.993** | **0.990** | **0.990** | **0.989** | **0.999** | 1 |  |
| PD3Y3 | **0.993** | **0.992** | **0.992** | **0.991** | **0.992** | **0.991** | **0.997** | **0.996** | 1 |

Values in bold are different from zero (P≤0.01). PD1, PD2 and PD3 are early abbreviations early, conventional and delayed planting dates, respectively. Y1, Y2 and Y3 are 2018/2019, 2019/2020 and 2020/2021, respectively

**Supplementary Table 4.** Cluster analysis for mean AUDPC trait of spot blotch.

| **Class** | **lines** | **Total** |
| --- | --- | --- |
| First Cluster | 1, 3, 4, 5, 6, 7, 8, 9, 10, 11, 12, 14, 17, 30, 40, 48, 54, 55, 57, 58, 65, 66, 70, 75, 77, 84, 91, 93, 94, 95, 97, 98, 99 | 33 |
| Second  cluster | 2, 13, 15, 20, 26, 29, 31, 32, 35, 43, 52, 60, 61, 62, 63, 64, 67, 68, 69, 73, 78, 79, 87, 89, 90, 96, 102 | 27 |
| Third cluster | 16, 18, 19, 21, 22, 23, 24, 25, 27, 28, 33, 34, 36, 37, 38, 39, 41, 42, 44, 45, 46, 47, 49, 50, 51, 53, 56, 59, 71, 72, 74, 76, 80, 81, 82, 83, 85, 86, 88, 92, 100, 101, 103 | 43 |

**Supplementary Table 5.** Mean AUDPC trait of spot blotch in different planting dates for each cluster

| Class | Mean |  | PD1 | | |  | PD2 | | |  | PD3 | | |
| --- | --- | --- | --- | --- | --- | --- | --- | --- | --- | --- | --- | --- | --- |
|  |  |  | 2018-2019 | 2019-2020 | 2020-2021 |  | 2018-2019 | 2019-2020 | 2020-2021 |  | 2018-2019 | 2019-2020 | 2020-2021 |
| First cluster | 573.89 |  | 765.23 | 766.25 | 764.82 |  | 557.25 | 552.36 | 561.45 |  | 401.85 | 397.53 | 398.24 |
| Second cluster | 467.05 |  | 642.25 | 643.83 | 643.83 |  | 453.70 | 445.26 | 451.92 |  | 305.26 | 306.79 | 310.58 |
| Third cluster | 347.11 |  | 522.84 | 526.54 | 526.87 |  | 306.79 | 305.39 | 303.99 |  | 210.22 | 210.75 | 210.71 |

PD1, PD2 and PD3 are early, conventional and delayed planting dates, respectively.

**Supplementary Table 6.** The osmotic potentials of flowering stage in 2018/2019, 2019/2020 and 2020/2021.

| **Drought Stress Levels** | | | **Planting Date** |
| --- | --- | --- | --- |
| **2020-2021** | **2019-2020** | **2018-2019** |  |
| -8.14 | -4.27 | -2.16 | Early |
| -8.97 | -6.11 | -4.45 | Conventional |
| -11.87 | -8.63 | -6.39 | Delayed |

The unit of drought levels (Soil Water Potential: SWP) is the Mega Pascal (MPa).


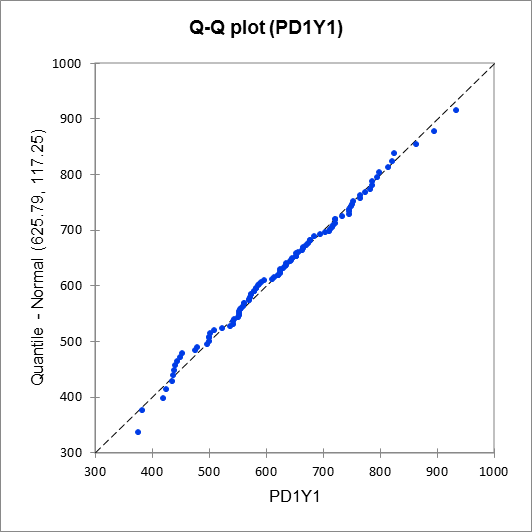


A


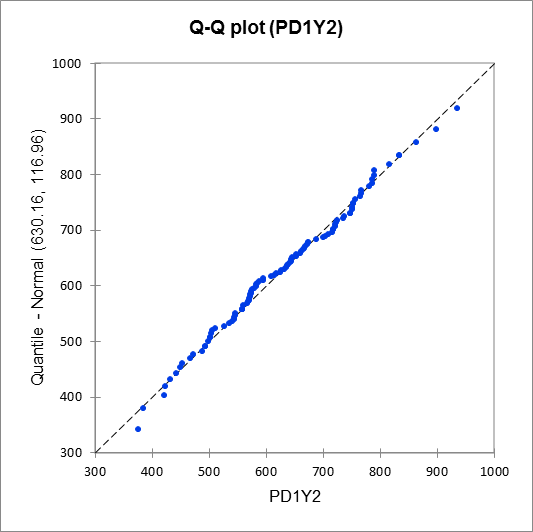


B


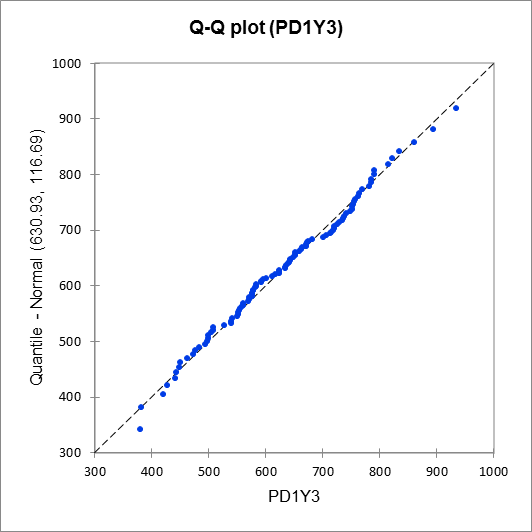


C

**Supplementary Figure 1.** Q-Q plot for AUDPC of spot blotch in the early planting date (PD1) in 2018/2019 (**A**), 2019/2020 (**B**) and 2020/2021 (**C**) (Y1-Y3, respectively).


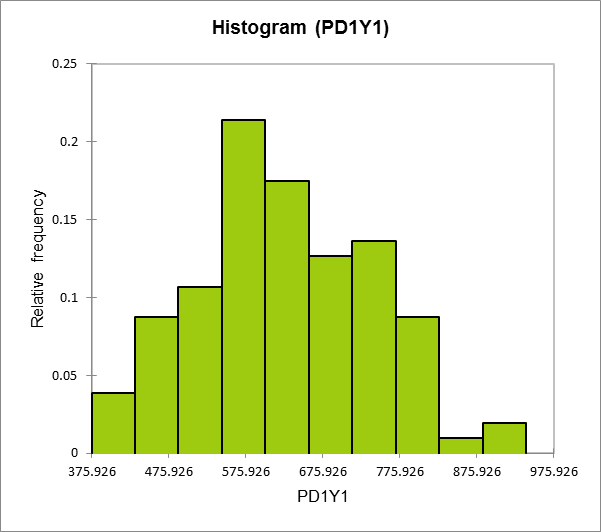


A


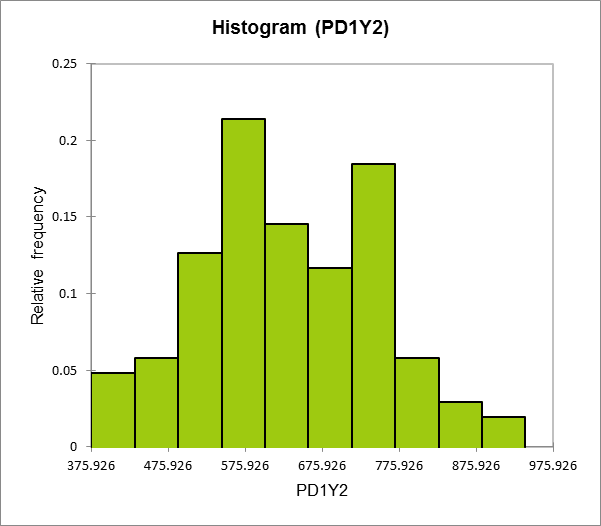


B


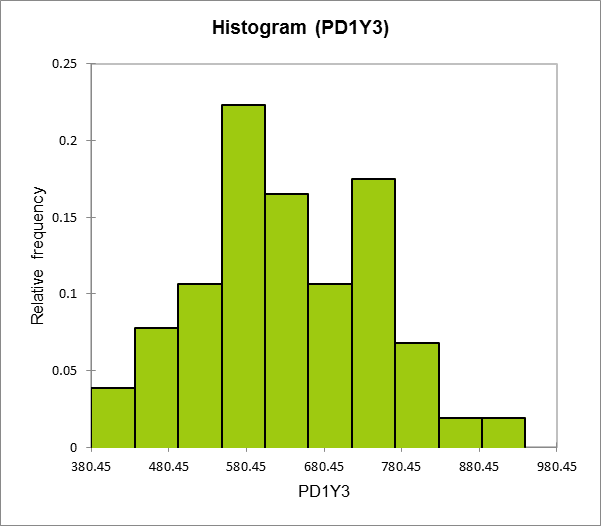


C

**Supplementary Figure 2.** Phenotypic distribution for AUDPC of spot blotch in the early planting date (PD1) in 2018/2019 (**A**), 2019/2020 (**B**) and 2020/2021 (**C**) (Y1-Y3, respectively).


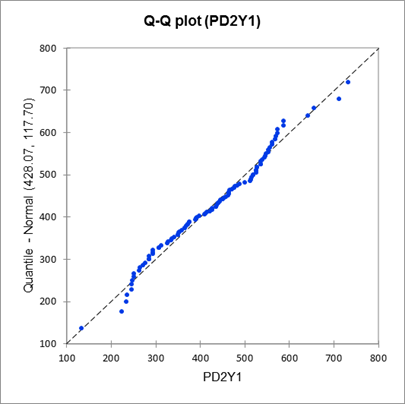


A


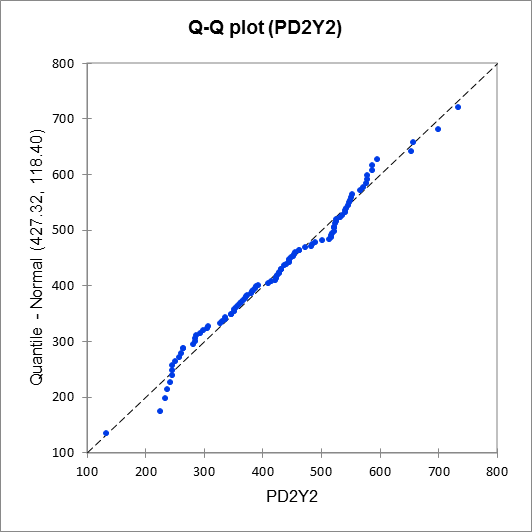


B


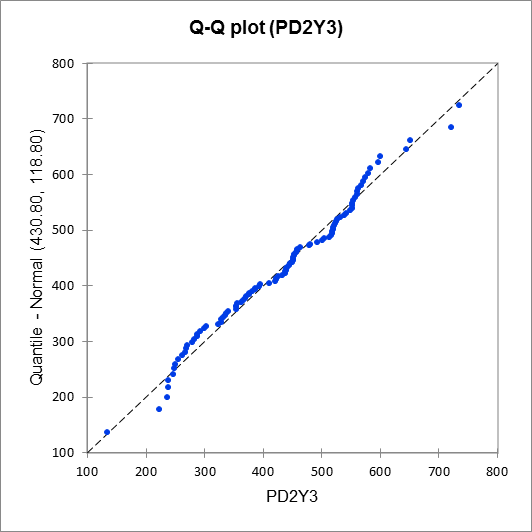


C

**Supplementary Figure 3.** Q-Q plot for AUDPC of spot blotch in the conventional planting date (PD2) in 2018/2019 (**A**), 2019/2020 (**B**) and 2020/2021 (**C**) (Y1-Y3, respectively).


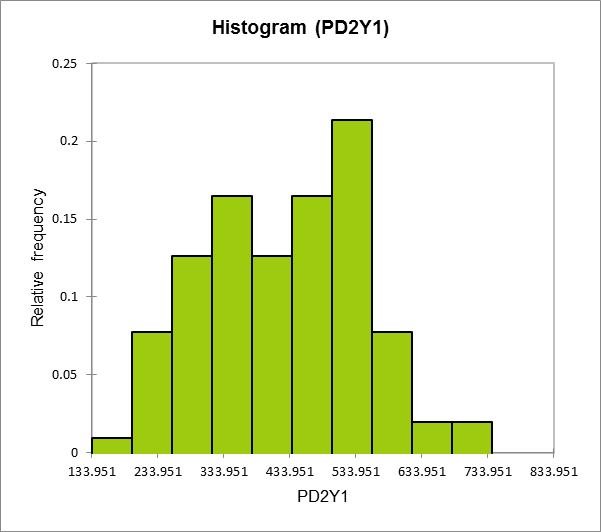


A


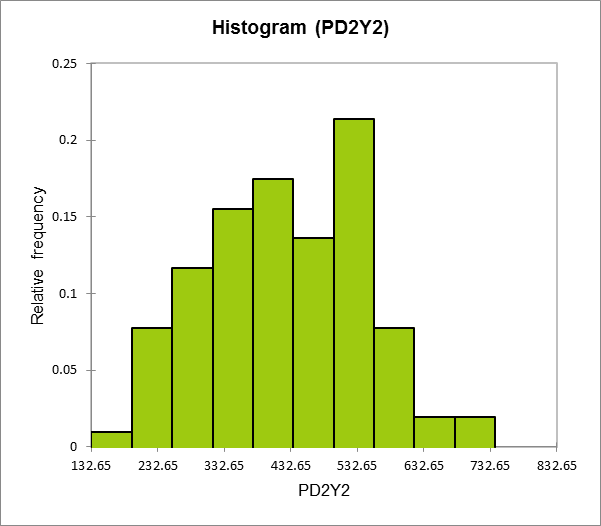


B


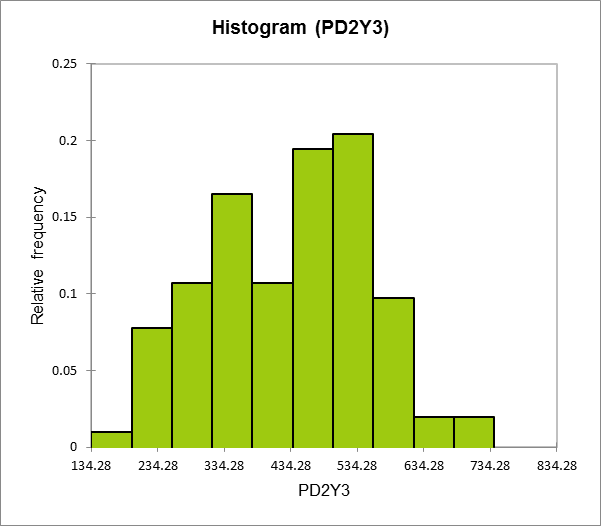


C

**Supplementary Figure 4.** Phenotypic distribution for AUDPC of spot blotch in the conventional planting date (PD2) in 2018/2019 (**A**), 2019/2020 (**B**) and 2020/2021 (**C**) (Y1-Y3, respectively).


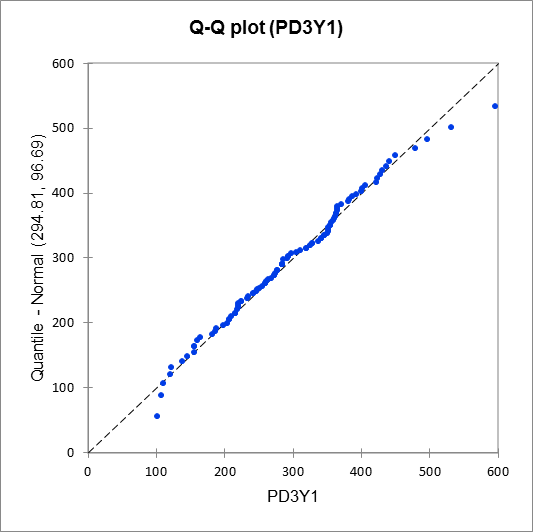


A


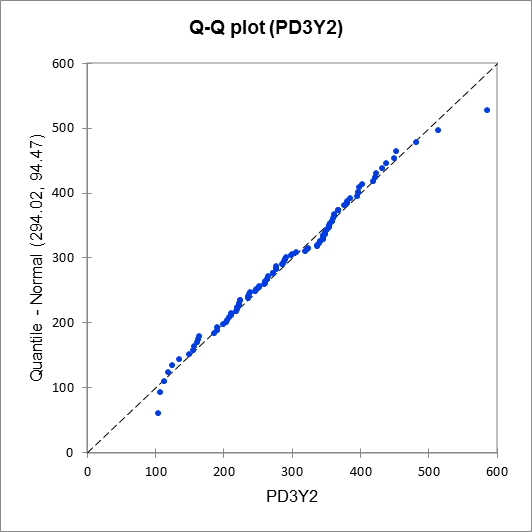


B


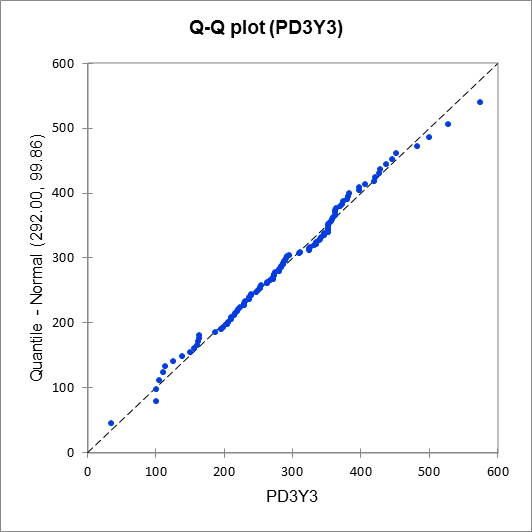


C

**Supplementary Figure 5.** Q-Q plot for AUDPC of spot blotch in the delayed planting date (PD3) in 2018/2019 (**A**), 2019/2020 (**B**) and 2020/2021 (**C**) (Y1-Y3, respectively).


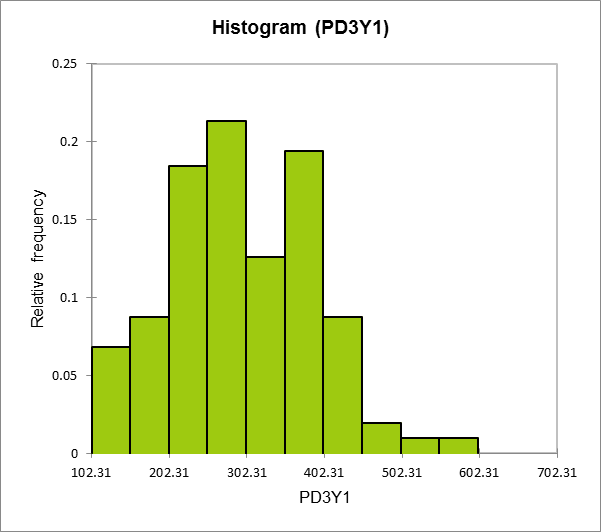


A


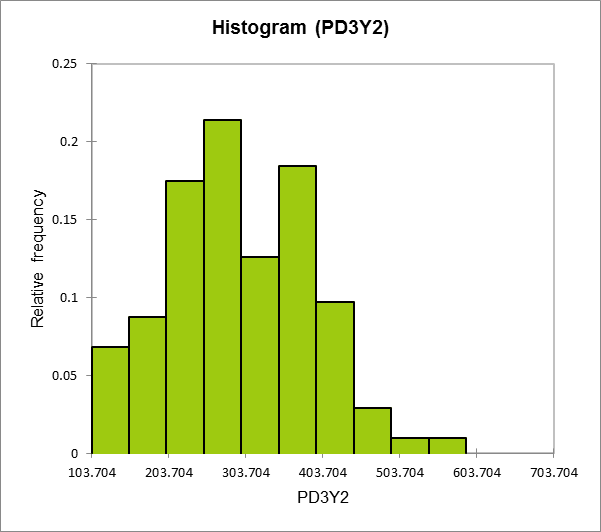


B


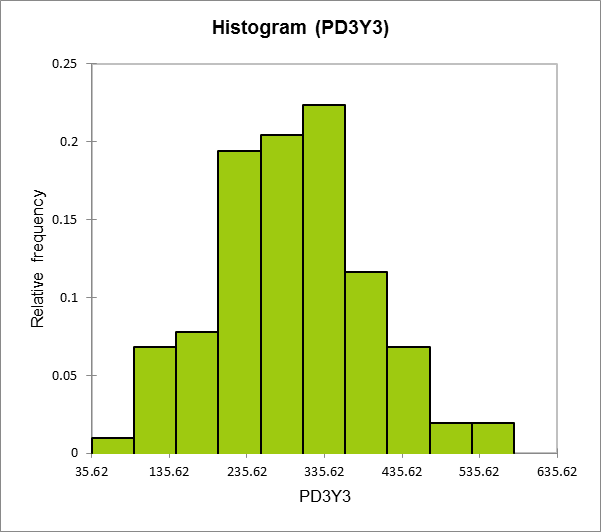


C

**Supplementary Figure 6.** Phenotypic distribution for AUDPC of spot blotch in the delayed planting date (PD3) in 2018/2019 (**A**), 2019/2020 (**B**) and 2020/2021 (**C**) (Y1-Y3, respectively).


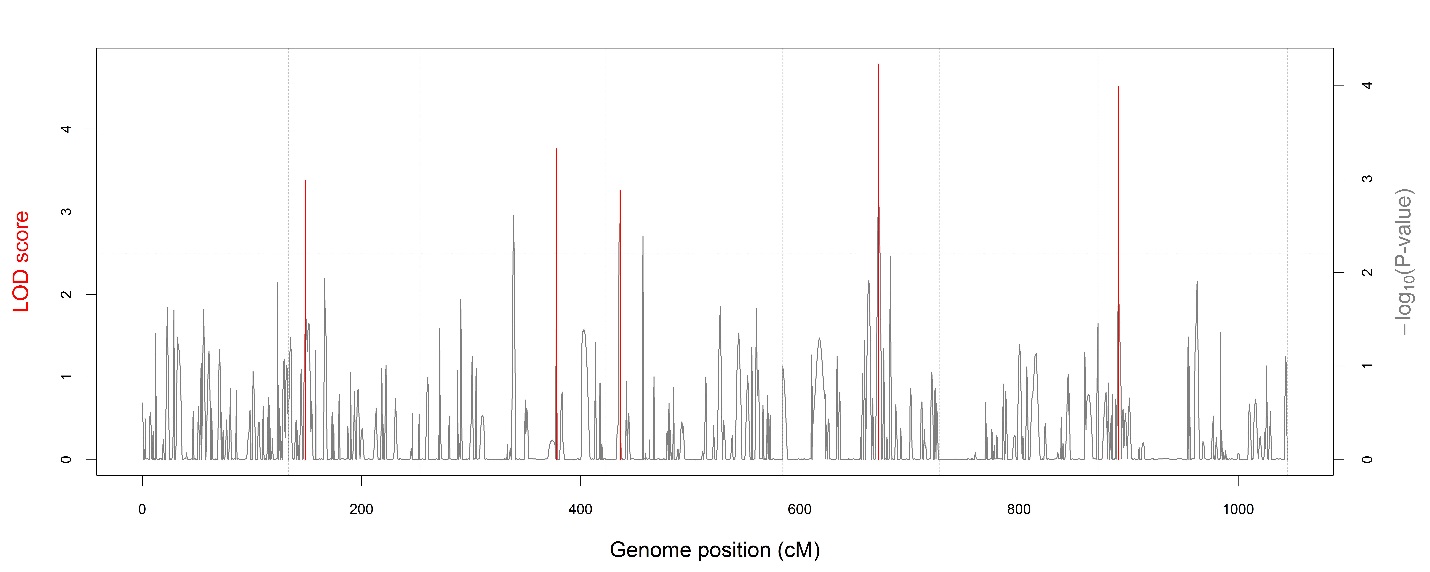


B

A


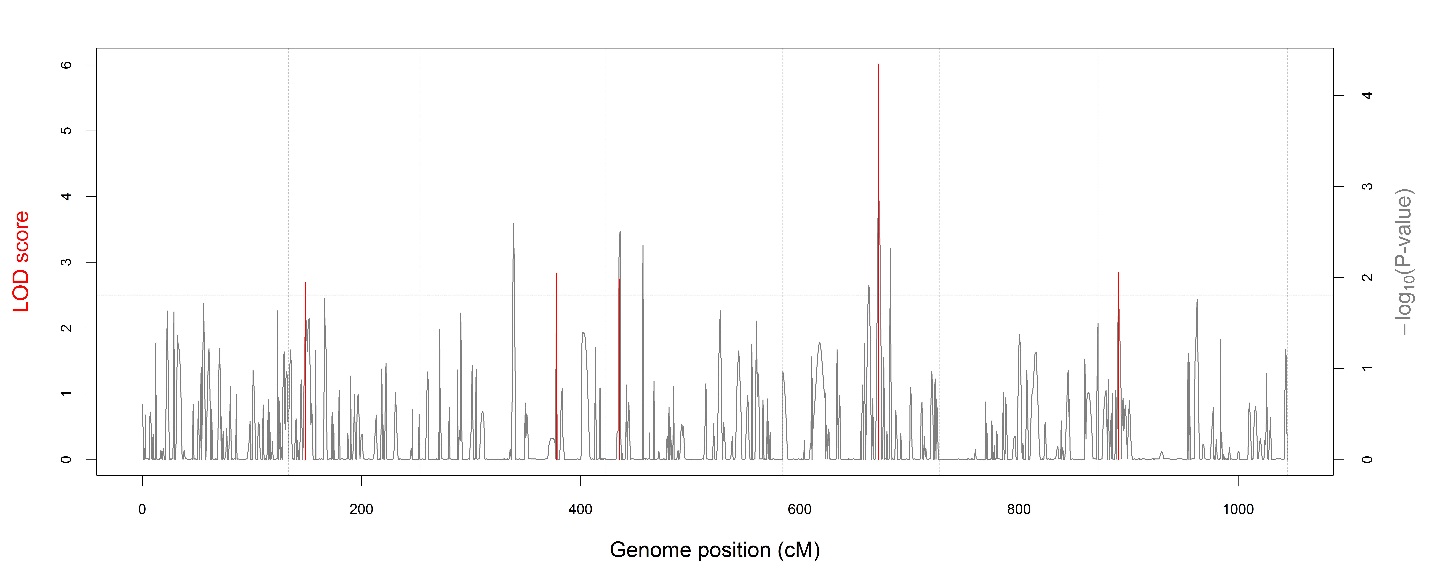


C


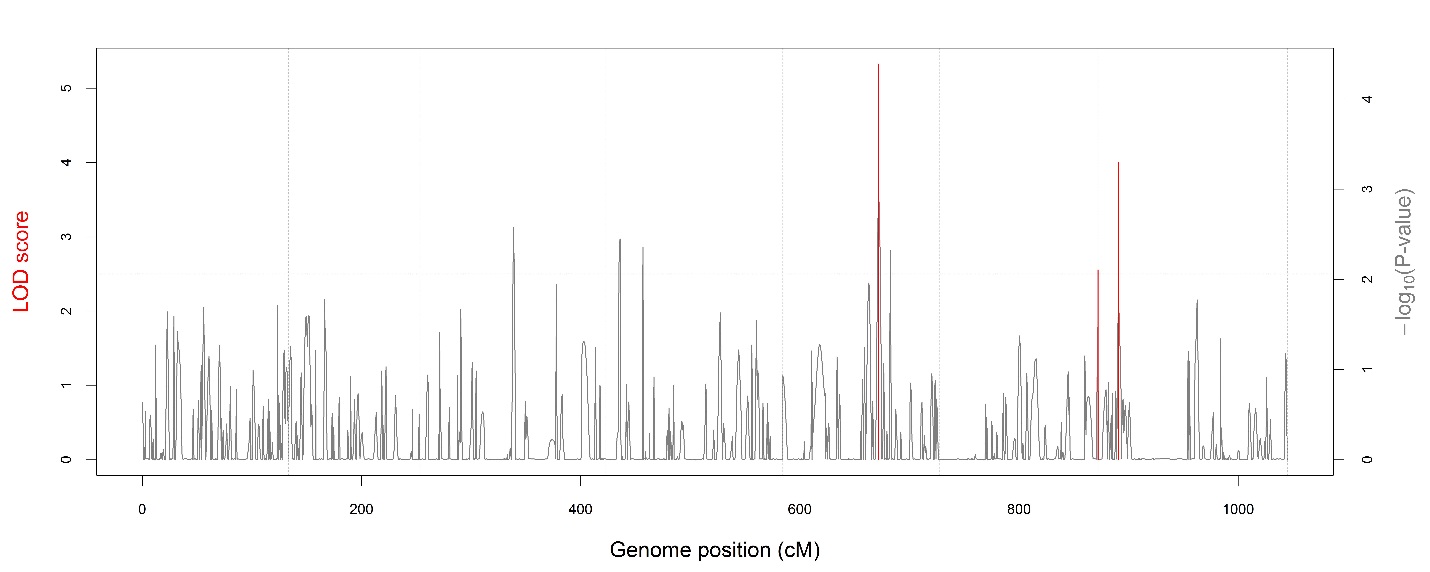


**Supplementary Figure 7.** Finding stable and closely linked QTLs of resistance/susceptibility against spot blotch in the early planting date in 2018/2019 (**A**), 2019/2020 (**B**) and 2020/2021 (**C**). LOD score>2.5 were significant and confirmed QTL. The confirmed QTL line were marked with red color.


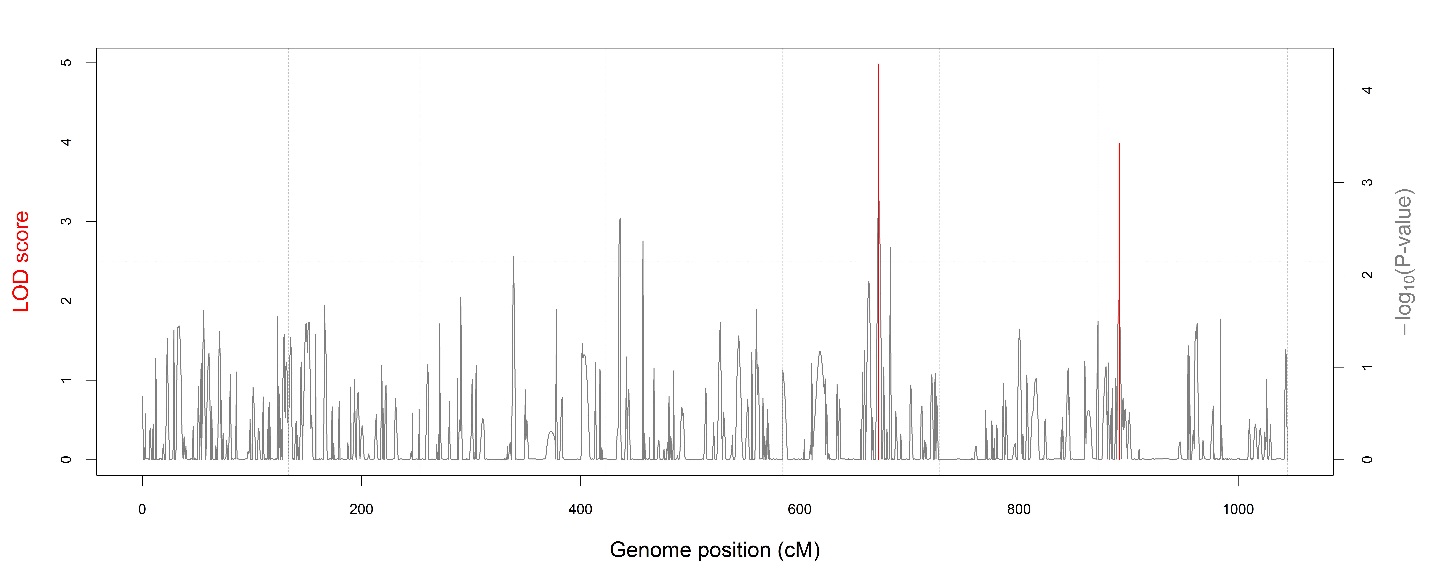


C

B

A


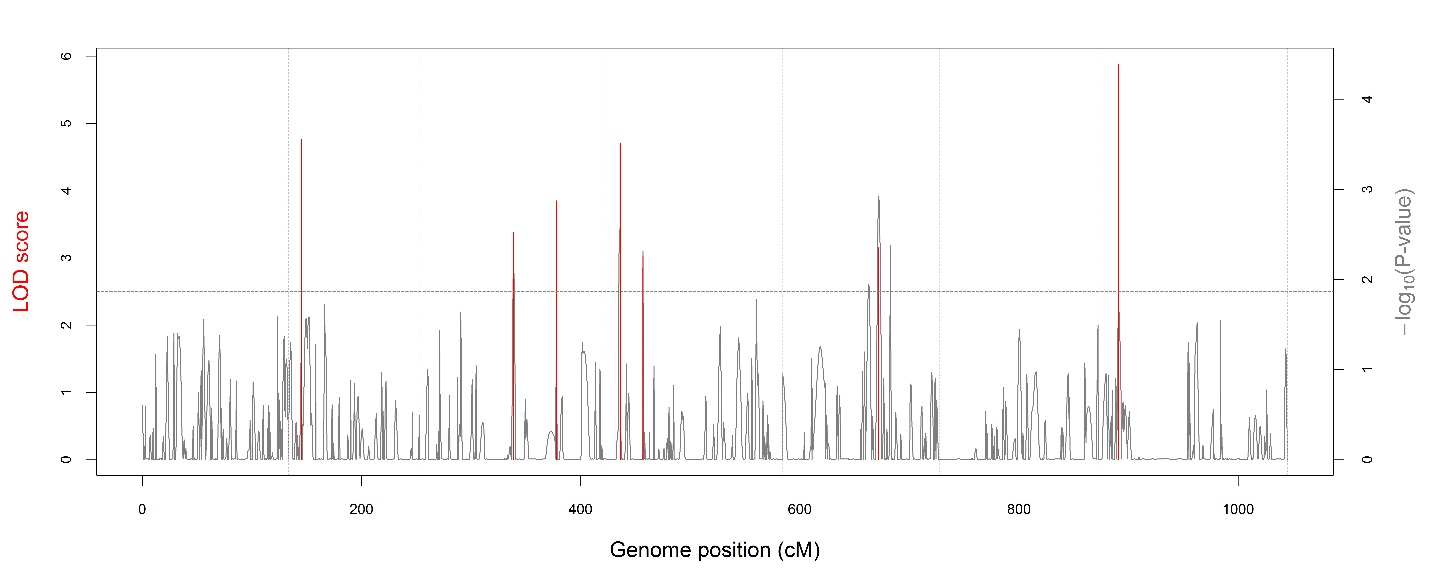

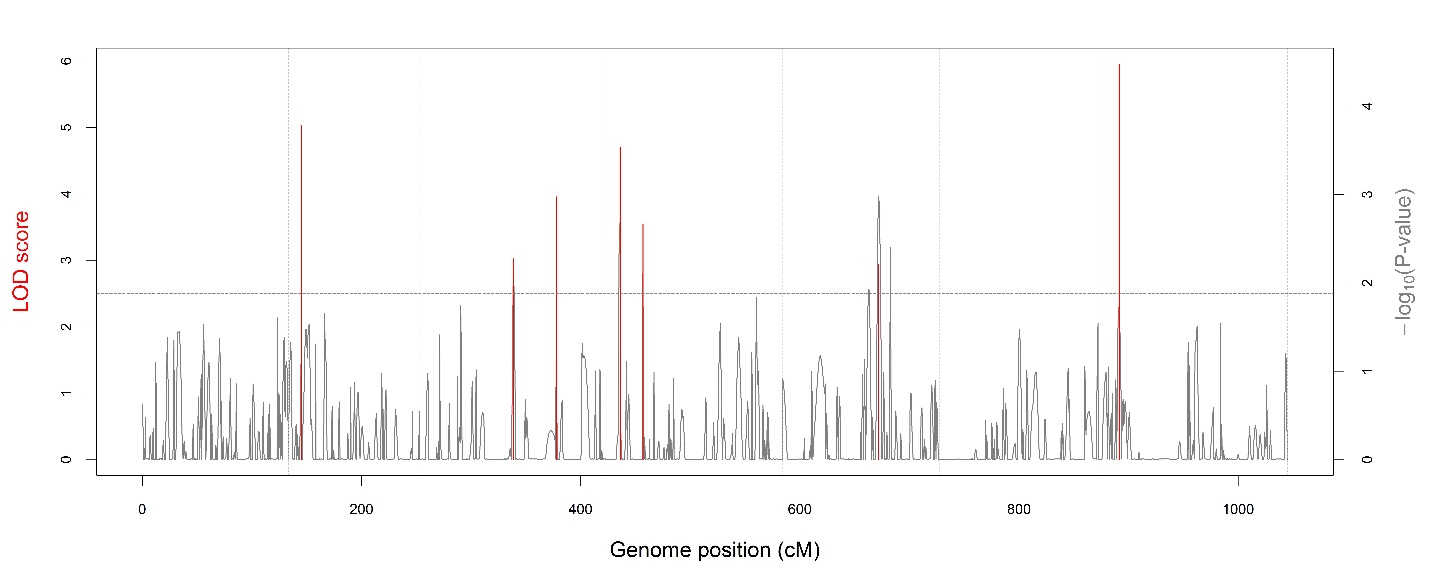


**Supplementary Figure 8.** Finding stable and closely linked QTLs of resistance/susceptibility against spot blotch in the conventional planting date in 2018/2019 (**A**), 2019/2020 (**B**) and 2020/2021 (**C**). LOD score>2.5 were significant and confirmed QTL. The confirmed QTL line were marked with red color.


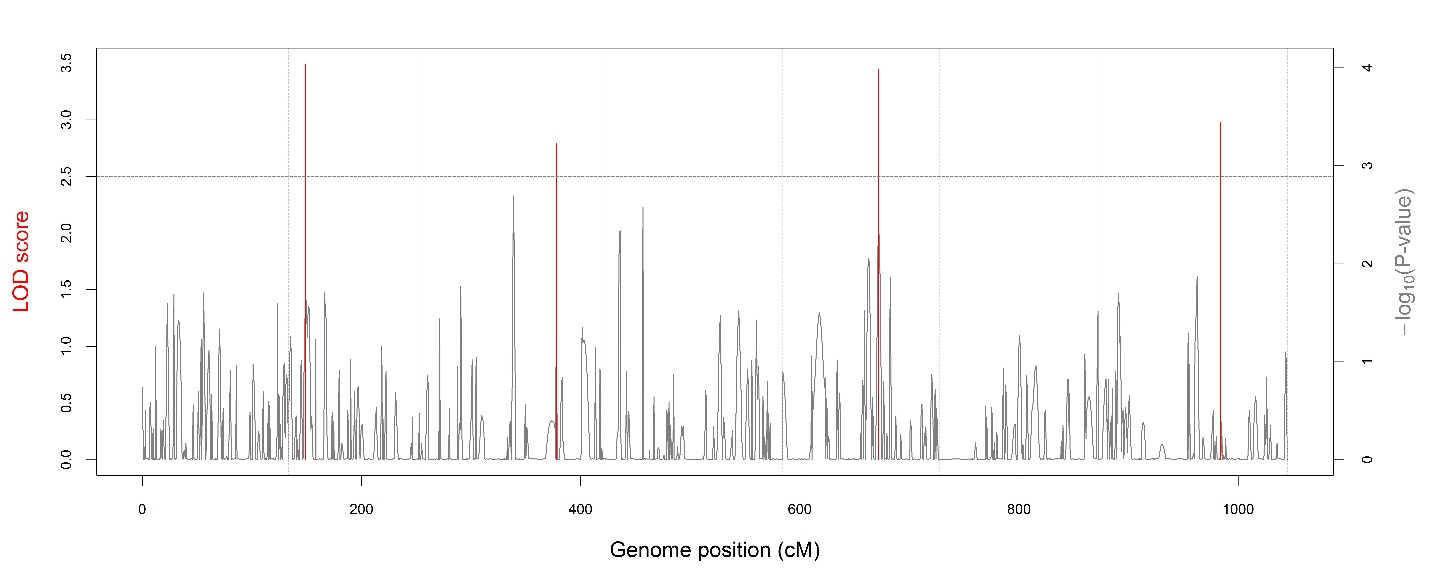


B

A


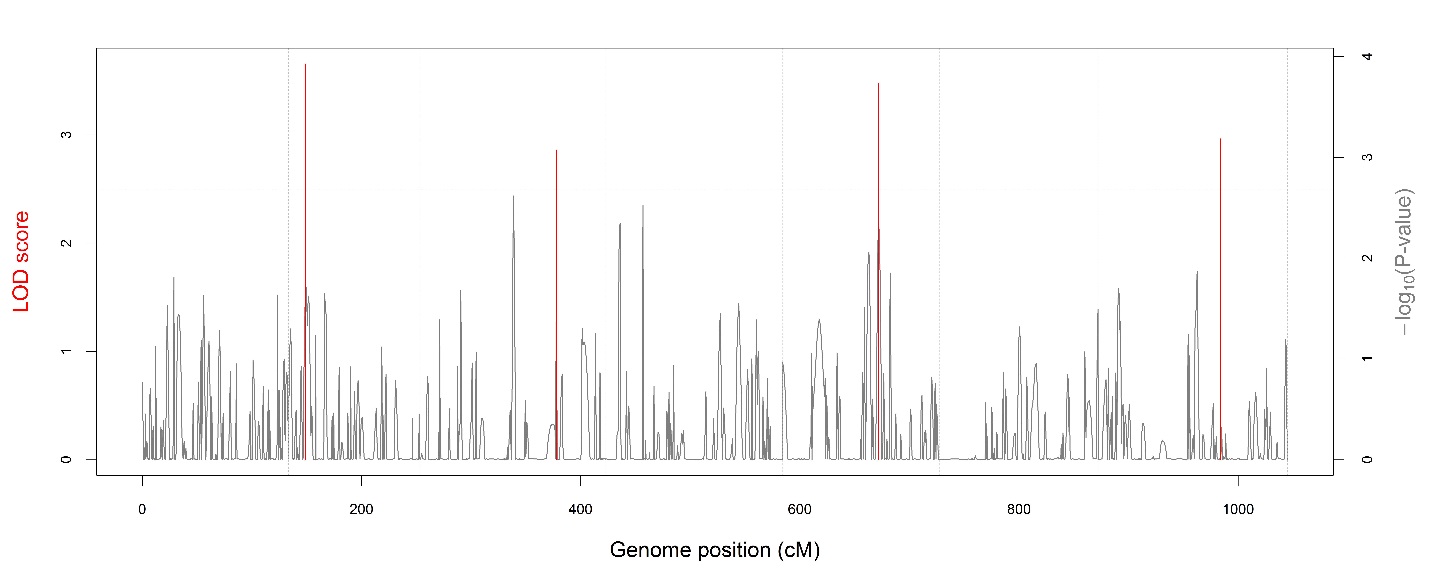


C

**Supplementary**
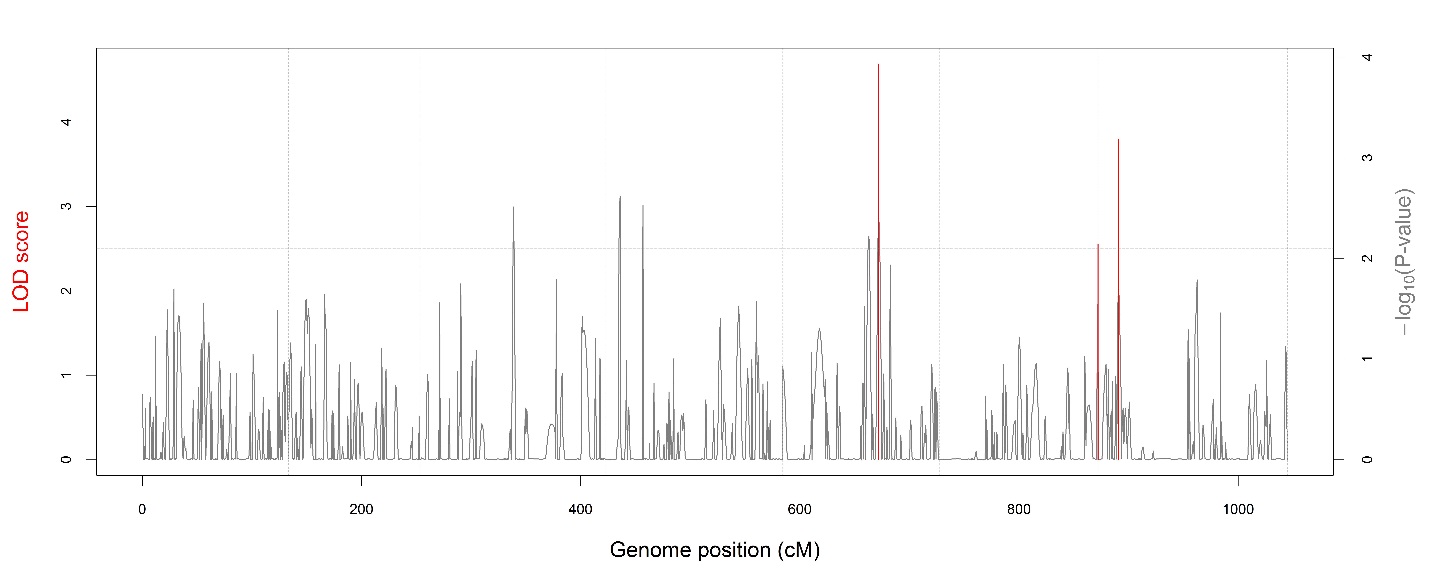
**Figure 9.** Finding stable and closely linked QTLs of resistance/susceptibility against spot blotch in the delayed planting date in 2018/2019 (**A**), 2019/2020 (**B**) and 2020/2021 (**C**). LOD score>2.5 were significant and confirmed QTL. The confirmed QTL line were marked with red color.
